# Perhydropolysilazane as a Precursor for Solution-Processed Silicon-Based Materials: Synthesis, Conversion Routes and Mechanisms, Properties, and Applications

**DOI:** 10.3390/polym18172113

**Published:** 2026-08-31

**Authors:** Su Jung Lee, Sangdeok Shim, Hyeon Mo Cho

**Affiliations:** 1 Research Institute for Liberal Education, University College, Yonsei University, Incheon 21983, Republic of Korea; 2 Department of Chemistry, Sunchon National University, Suncheon 57922, Republic of Korea; san90@scnu.ac.kr; 3 University College, Yonsei University, Incheon 21983, Republic of Korea

**Keywords:** perhydropolysilazane (PHPS), inorganic polymer, solution processing, silicon-based materials, silica coating, silicon oxynitride, silicon nitride, structure–property relationships

## Abstract

Perhydropolysilazane (PHPS) has attracted considerable attention as a versatile precursor for solution-processed silicon-based materials because of its carbon-free backbone, high chemical reactivity, and compatibility with low-temperature processing. This review provides a comprehensive overview of the synthesis of PHPS, its conversion routes and mechanisms, the structure–property relationships of PHPS-derived materials, and their current and emerging applications. The literature demonstrates that PHPS conversion is governed by coupled hydrolysis, oxidation, nitridation, dehydrogenation, photochemical activation, and reactive-species-mediated processes, resulting in compositionally and structurally diverse silicon-based networks, including SiO_x_-rich, SiO_x_Nᵧ, and SiN_x_ materials. The resulting optical, mechanical, surface, and barrier properties are determined by network structure, residual bonding environments, compositional gradients, and interfacial characteristics rather than by the nominal conversion method alone. Recent advances further demonstrate the potential of PHPS-derived materials for barrier coatings, dielectric and interfacial layers, protective coatings, semiconductor processing, and high-temperature ceramic applications. Overall, PHPS should be regarded not merely as a silica precursor but as a tunable preceramic platform for engineering diverse silicon-based networks. Future progress will depend on predictive control of conversion processes, standardized evaluation protocols, sustainable solution-based manufacturing, and comprehensive device-level validation to facilitate practical implementation.

## 1. Introduction

**Figure 1:**
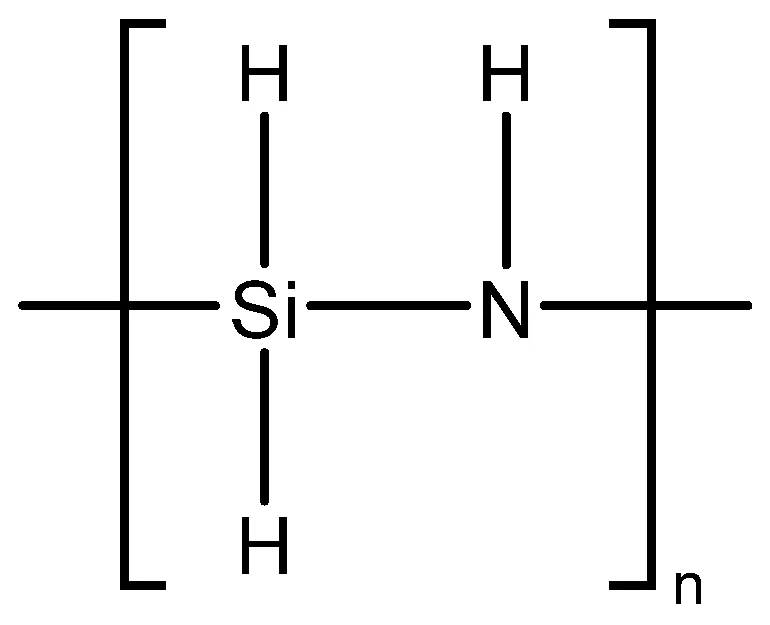
Chemical structure of perhydropolysilazane (PHPS).

Perhydropolysilazane (PHPS) is an inorganic polysilazane composed exclusively of silicon, nitrogen, and hydrogen atoms, with a molecular structure consisting solely of Si–N, Si–H, and N–H bonds. It is generally represented by a repeating –[SiH_2_–NH]–_n_ backbone and is distinguished from organopolysilazanes by the absence of carbon-containing substituents (Figure 1) [1,2,3]. The high reactivity of the Si–N and Si–H bonds allows PHPS to readily undergo chemical conversion upon exposure to moisture, oxygen, ultraviolet irradiation, or thermal treatment, yielding a variety of silicon-based inorganic materials. A notable advantage of PHPS is its ability to form silica thin films under ambient or relatively low-temperature conditions, distinguishing it from conventional silica fabrication routes based on tetraalkoxysilane precursors. This unique conversion behavior has attracted considerable attention for the preparation of silicon-based ceramic coatings, barrier layers, and electronic materials [1,2,3,4,5,6,7,8].

Unlike modified polysilazanes containing alkyl or aryl substituents, PHPS possesses a carbon-free backbone. This structural feature is particularly significant because the absence of carbon-containing substituents minimizes carbon incorporation during conversion, thereby enabling the formation of high-purity silicon oxide, silicon oxynitride, and silicon nitride networks [2,7,8]. In contrast, organic substituents may influence the conversion behavior through steric effects, the retention of Si–C bonds, and alterations in crosslinking pathways, often affecting the composition and microstructure of the resulting ceramic materials [4,5,9,10].

The conversion of PHPS is understood as a network reconstruction process involving the cleavage, substitution, oxidation, condensation, dehydrogenation, and rearrangement of Si–H and Si–N bonds. Depending on the reaction atmosphere and activation method, PHPS is transformed into SiO_2_-rich, SiO_x_Nᵧ, or SiN_x_-based networks [2]. Under humid conditions or in NH_3_/H_2_O-containing environments, hydrolysis and condensation reactions promote the formation of Si–O–Si networks [11,12]. In contrast, oxygen-deficient or nitrogen-rich conditions favor the retention or reconstruction of Si–N-based bonding environments [7]. Furthermore, external activation methods, including catalysts, radical species, and UV/VUV (vacuum ultraviolet) irradiation, enhance the conversion kinetics by promoting bond cleavage, oxygen incorporation, nitrogen elimination, and network densification, thereby enabling control over the composition and microstructure of the resulting ceramic materials [3,8,13].

PHPS-derived materials have been widely utilized in various industrial fields owing to their excellent transparency, low surface roughness, high thermal stability, environmental stability, and outstanding gas barrier properties. In particular, PHPS-derived silicon oxide and silicon oxynitride coatings exhibit high optical transparency and dense microstructures, making them attractive for protective coatings, barrier layers, and electronic materials [14,15,16,17].

Another significant advantage of PHPS in ceramic thin-film fabrication is its excellent solubility in organic solvents. PHPS readily dissolves in various organic solvents, such as xylene and dibutyl ether, enabling the preparation of homogeneous coating solutions. This characteristic facilitates the application of a wide range of solution-based processes, including spin coating, dip coating, and inkjet printing, while also allowing the formation of uniform thin films over large-area substrates. Compared with conventional vapor-phase deposition techniques, such as chemical vapor deposition (CVD), physical vapor deposition, and atomic layer deposition (ALD), solution-based processing significantly reduces equipment costs and energy consumption. In addition, PHPS-based coating processes exhibit excellent compatibility with roll-to-roll manufacturing. Therefore, the high solubility of PHPS in organic solvents represents a key advantage for the low-cost and large-area fabrication of ceramic thin films [12,18,19,20].

More recently, PHPS-derived silica materials have been employed as dielectric-forming materials in advanced semiconductor manufacturing processes [21,22,23]. The formation of silica layers for shallow trench isolation (STI) using PHPS offers several advantages over conventional CVD-based approaches. With the excellent flowability and penetration capability of the liquid precursor, PHPS enables more uniform filling of high-aspect-ratio trenches. As a result, the formation of voids and seams during the filling process is effectively suppressed, leading to improved dielectric performance and device reliability. In addition, PHPS is readily converted into high-quality SiO_2_ films at relatively low temperatures, thereby reducing the thermal budget of the process. Unlike plasma-assisted deposition methods, PHPS-based processes minimize plasma-induced damage to substrates and interfaces. Notably, the excellent self-planarization behavior of PHPS-derived films alleviates the burden of subsequent chemical mechanical polishing processes. These advantages provide important benefits for improving dielectric quality and ensuring process stability in the semiconductor industry, where device integration density and miniaturization continue to advance.

In addition, PHPS has attracted considerable attention as a ceramic coating precursor for protecting steel surfaces exposed to the harsh environments encountered in the oil and gas industry, including high temperatures, high pressures, and corrosive media. Its excellent chemical stability and corrosion resistance contribute to improved durability and extended service life of industrial facilities and components [24,25]. PHPS has also been explored for aerospace applications through the fabrication of silica- and silicon-based ceramic coatings that combine lightweight characteristics with excellent thermal stability. These coatings provide effective protection of structural materials against thermal degradation and environmental damage under extreme operating conditions [26,27]. In particular, PHPS-derived silica coatings have demonstrated excellent resistance to atomic oxygen attack, highlighting their potential for spacecraft and aerospace material protection. PHPS-derived coatings have also shown considerable potential for automotive applications as surface treatment technologies for enhancing the corrosion and wear resistance of metallic components and improving their long-term reliability. The excellent tribocorrosion resistance and protective performance of PHPS-derived ceramic coatings make them attractive candidates for extending the service lifetime of steel-based automotive components operating under demanding environments [28]. As a result of these advantageous properties, PHPS is widely regarded as a promising precursor for a broad range of industrial protective coatings designed for operation in harsh and extreme environments.

Given its unique chemical structure and excellent inorganic conversion characteristics, PHPS has emerged as a key precursor for the fabrication of silica- and silicon-based ceramic materials. Despite the availability of several review articles addressing polysilazane-based materials and polymer-derived ceramics, reviews specifically focused on PHPS remain limited. Therefore, this review provides a comprehensive overview of the historical development and synthesis methodologies of PHPS, its conversion reactions and underlying mechanisms, and the properties of PHPS-derived silica and silicon-based ceramic materials. The current and emerging applications of PHPS-derived materials are also systematically summarized. Finally, recent research trends are examined to identify the key scientific and technical challenges in PHPS conversion and application, while highlighting future directions toward predictive processing, sustainable manufacturing, and practical implementation.

## 2. Synthesis of PHPS

In 1921, Stock and Somieski reported what is generally considered the first synthesis of a PHPS-related polysilazane material. By reacting dichlorosilane (DCS, H_2_SiCl_2_) with NH_3_ in a benzene solvent, they obtained a volatile oily product [29]. However, the material was unstable at room temperature and gradually transformed into a hard glassy solid after approximately 24 h. According to Seyferth et al., the products obtained in this early study were oligomeric species with a molecular weight of approximately 350, and no substantial follow-up investigations were conducted for several decades thereafter [30]. Meanwhile, in 1966, Campbell-Ferguson and Ebsworth reported the formation of adducts between halosilanes and organic bases. Specifically, they demonstrated that DCS forms a 1:2 adduct with excess pyridine [31]. This finding later provided an important foundation for the synthesis of high-molecular-weight PHPS. Subsequently, in 1998, Hensen et al. determined the solid-state structure of *trans*-SiH_2_Cl_2_(pyridine)_2_ by single-crystal X-ray diffraction, confirming a hexacoordinate structure involving two Si–N bonds [32]. In addition, Scantlin and Norman investigated the borane-catalyzed condensation of trisilazane ((SiH_3_)_3_N) in 1972 and proposed the importance of silazane–borane complexation (adduct formation) during the early stages of the reaction [33].

In 1983, Seyferth and co-workers reported a significant advance in the development of PHPS as a ceramic precursor [30]. PHPS oil was synthesized by reacting DCS with gaseous ammonia in polar solvents such as dichloromethane or diethyl ether. The PHPS oil was subsequently pyrolyzed under a nitrogen atmosphere at temperatures up to 1150 °C, yielding silicon nitride ceramics with a ceramic yield of approximately 70%. This study confirmed the feasibility of converting PHPS into silicon nitride ceramics and established PHPS as a promising precursor for polymer-derived ceramic materials. However, the PHPS obtained at that time still exhibited limited storage stability, as evidenced by a gradual increase in viscosity even at room temperature.

**Figure 2:**
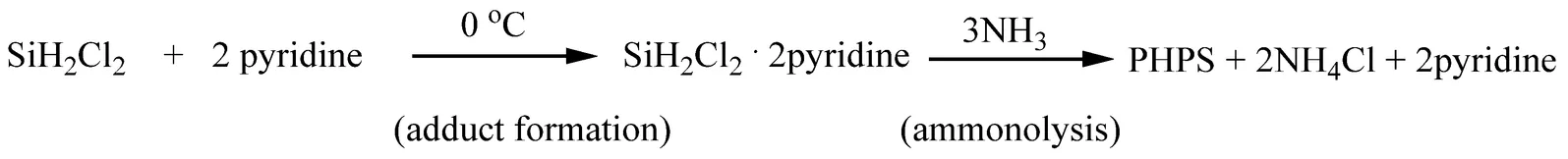
Synthetic method of PHPS.

In 1985, Isoda and Arai successfully synthesized polymeric PHPS through the ammonolysis of a DCS–pyridine adduct (Figure 2) [34]. This work represented a major breakthrough in PHPS synthesis, and the stability of the DCS–pyridine adduct was considered to play a crucial role in enabling the preparation of high-molecular-weight polymers. PHPS prepared by this method was readily processed into continuous fibers and subsequently converted into high-purity silicon nitride fibers with excellent ceramic yields. They further demonstrated that pyridine not only forms a stable adduct with DCS but also promotes dehydrogenative condensation and disproportionation reactions during subsequent heat treatment. These reactions contribute to the control of molecular weight and the development of branching structures within the polymer. Oligomeric PHPS synthesized at 0 °C exhibited molecular weights of approximately 700 (Mn) and 1000 (Mw). Subsequent heat treatment in a pyridine solution at 120 °C for 4 h increased the molecular weight to approximately 1400 (Mn) and 4370 (Mw), resulting in the formation of a highly branched polymer [35]. The ceramic yield of the resulting PHPS ranged from 82 to 93% and was strongly dependent on the degree of branching within the polymer structure. Specifically, the ceramic yield increased with increasing branching parameter, which reflects the content of branching units such as −SiH< and −N< groups. The synthetic strategy involving the initial formation of a DCS–pyridine adduct followed by ammonolysis with ammonia has since become one of the most widely adopted routes for the preparation of PHPS. Due to the inherent instability of Si-N bonds toward hydrolysis and the high moisture sensitivity of PHPS, all experimental procedures—including synthesis, heat treatment, and isolation—must be strictly conducted under a dry inert atmosphere of N_2_ or Ar [30,35].

## 3. Conversion Methods of PHPS

This section reviews the methods used to convert PHPS into SiN-, SiO-, and SiON-based materials, while providing a brief overview of the corresponding reaction pathways and resulting material properties. Detailed discussions of the conversion mechanisms and material properties are presented in Section 4 and Section 5, respectively.

### 3.1. Preparation of SiN-Based Materials

Methods for the preparation of silicon nitride (SiN_x_) materials using PHPS as a precursor have primarily involved photochemical curing induced by VUV irradiation and pyrolytic conversion through thermal treatment. These conversion processes are generally conducted under inert atmospheres to suppress oxidation and promote the formation of Si–N-based ceramic networks. For applications requiring thin protective coatings, VUV-assisted curing is generally preferred because it enables the conversion of PHPS at relatively low temperatures, thereby minimizing thermal damage to substrates while allowing rapid processing. In contrast, pyrolytic conversion has been widely employed for the fabrication of high-strength structural materials, as it allows control over the degree of densification and crystallinity of the resulting silicon nitride ceramics. Accordingly, the selection of the conversion route is largely determined by the intended application and the desired material properties.

#### 3.1.1. Photochemical Curing via VUV Irradiation

Photochemical curing through VUV irradiation has been primarily employed for the fabrication of gas barrier layers for flexible electronic devices that are sensitive to thermal processing, such as organic light-emitting diodes (OLEDs) and perovskite solar cells (PSCs) [36,37,38].

PHPS exhibits strong photon absorption in the VUV region, with absorption increasing sharply as the wavelength decreases from 210 to 164 nm, making 172 nm VUV irradiation particularly suitable for photochemical curing [8]. VUV curing generally proceeds through photo-induced dehydrogenation followed by photo-densification. These processes lead to the formation of a dense Si–N network and an increase in film density, as evidenced by the increase in refractive index without a significant change in the film composition [36,37,38,39]. The detailed mechanism of VUV-induced conversion is discussed in Section 4.2. Because the presence of oxygen or moisture during the curing process can lead to the formation of Si–O-containing species, VUV curing is typically performed under a nitrogen atmosphere to obtain high-purity SiN-based materials [13,36,39].

**Figure 3:**
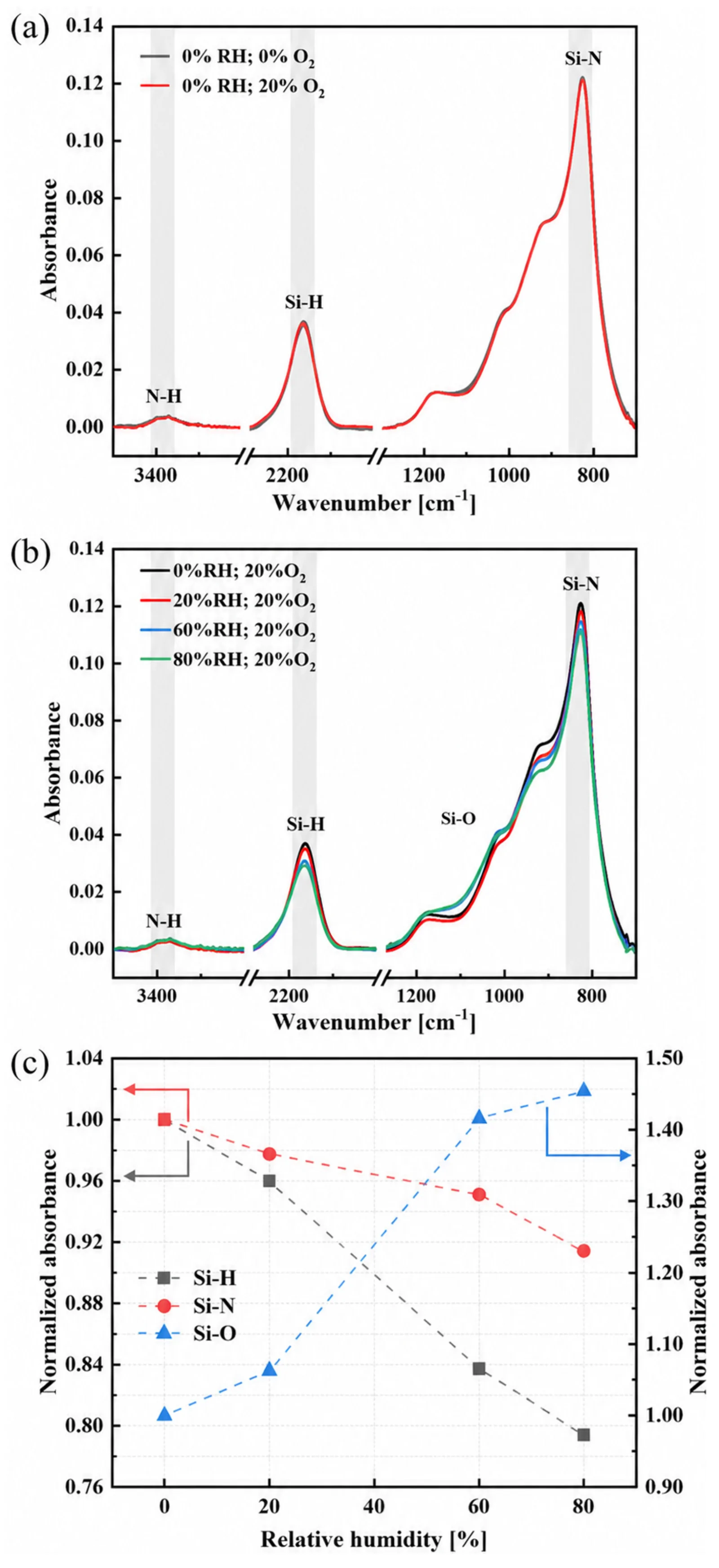
FTIR spectra of PHPS films prepared under different oxygen and relative humidity conditions after VUV irradiation at a dose of 6000 mJ cm^−2^. (**a**) Films processed at 0% relative humidity under 0% and 20% O_2_. (**b**) Films processed under 20% O_2_ at relative humidities of 0–80%. (**c**) Normalized Si–H, Si–N, and Si–O absorbance as a function of relative humidity. Adapted with permission from Ref. [36]. Copyright 2025, The Author(s).

Although both oxygen and moisture contribute to Si–O formation during PHPS conversion, moisture has been reported to play the dominant role. During VUV curing, increasing the relative humidity from 0% to 60% resulted in a decrease in the refractive index from 1.73 to 1.64, indicating a reduction in film density (Figure 3) [36,38]. In general, SiN_x_ films are known to possess higher densities than SiO_x_ films [13]. Therefore, the formation of Si–O bonds leads to a decrease in film density, which can adversely affect the gas and moisture barrier performance of the resulting coating. Moisture permeability is strongly influenced by the presence of free volume within the barrier layer. When free-volume elements larger than a water molecule (approximately 0.38 nm) exist within the film, moisture penetration becomes more facile. VUV irradiation effectively reduces free volume through atomic rearrangement within the Si–N network. In contrast, the incorporation of Si–O bonds tends to produce a relatively less dense network structure, thereby providing diffusion pathways for moisture transport and consequently reducing barrier performance [13].

The dehydrogenation process primarily exhibits the characteristics of a single-photon reaction and is therefore governed mainly by the total irradiation dose. In contrast, the densification process is highly sensitive to the irradiation intensity. Higher VUV intensities enhance Si–N bond cleavage and promote a greater reduction in free volume. In particular, the use of high-power VUV irradiation (>290 mW cm^−2^) facilitates repeated cleavage and reformation of Si–N bonds, enabling the formation of highly dense films even under ultrafast processing conditions. Under optimized VUV curing conditions, such as an irradiation dose of 6 J cm^−2^, the resulting SiN_x_ films exhibited refractive indices exceeding 1.74 and demonstrated excellent moisture barrier performance [13,37,38]. Furthermore, prolonged VUV exposure was reported to increase the refractive index to as high as 1.88, indicating further densification of the SiN_x_ network [39].

#### 3.1.2. Pyrolytic Conversion via Thermal Treatment

Pyrolytic conversion has been widely employed for the fabrication of ceramic fibers and nanocomposites. The specific processing conditions vary depending on the pyrolysis temperature and the presence or absence of catalysts [40,41,42]. When PHPS fibers are heat-treated under a nitrogen atmosphere, significant changes occur in both the structure and tensile strength of the resulting silicon nitride materials as a function of temperature. Funayama and coworkers reported that inorganic conversion begins at temperatures above approximately 900 °C, while the amorphous phase gradually transforms into crystalline α-Si_3_N_4_ in the temperature range of 1200–1400 °C [40]. The tensile strength of the fibers reaches its maximum value in the amorphous state immediately prior to crystallization. However, a pronounced decrease in tensile strength is observed following the onset of crystallization.

### 3.2. Preparation of SiO-Based Materials

#### 3.2.1. Thermal Curing and Pyrolysis

Thermal curing is the most widely employed method for converting PHPS into silica. When PHPS is heated in air, it reacts with atmospheric oxygen and moisture, releasing ammonia and hydrogen while incorporating oxygen into the network to form Si–O bonds [43,44]. Complete conversion of PHPS to silica is achieved by heat treatment at 600 °C for 30 min, as evidenced by the complete disappearance of the Si–H (2160 cm^−1^) and Si–N (840 cm^−1^) absorption bands and the exclusive presence of the Si–O band at approximately 1070 cm^−1^ in the Fourier-transform infrared spectroscopy (FTIR) spectrum [8,17,45].

After curing at 1000 °C, the films exhibit density, refractive index, and Young’s modulus comparable to those of bulk silica glass [12,46]. These findings highlight a key advantage of PHPS-derived silica: high-quality silica materials can be deposited or introduced into desired structures through simple and cost-effective solution-based processing routes.

In the presence of moisture, the conversion of PHPS proceeds at relatively low temperatures of 100–300 °C [46]. Water vapor promotes the hydrolysis of Si–H and Si–N bonds, leading to the formation of silanol (Si–OH) species, which subsequently undergo condensation reactions to generate siloxane (Si–O–Si) networks [3,47]. However, at temperatures below approximately 200 °C, the thermal energy is often insufficient to completely cleave the reactive bonds, resulting in incomplete conversion accompanied by residual nitrogen- and hydrogen-containing species within the film. To overcome this limitation and complete the hydrolysis-driven conversion process, a short UV photo-curing step has been proposed as a post-treatment approach [46].

Thermal curing produces highly dense silica films; however, exposure to elevated temperatures often results in substantial volumetric shrinkage of approximately 20%, primarily due to dehydroxylation reactions of residual silanol groups and continued network densification during the conversion process [6,11,21,48]. Such shrinkage is considered one of the major factors responsible for the formation of cracks and other structural defects within the film. Naganuma et al. reported that high-temperature thermal annealing resulted in a significant thickness reduction of approximately 19.5% relative to the dried precursor film, whereas VUV irradiation induced a much smaller contraction of only 3.4% [6]. These results indicate that although thermal curing is highly effective for achieving dense silica networks, the associated shrinkage may impose limitations on film integrity and dimensional stability.

#### 3.2.2. Photochemical Curing

As discussed above, thermal curing has inherent limitations when applied to thermally sensitive polymer substrates, such as poly(ethylene terephthalate) (PET) and poly(ethylene naphthalate) (PEN). Consequently, considerable efforts have been devoted to the development of photochemical curing approaches for the conversion of PHPS. Among these approaches, curing using high-power VUV (172 nm) irradiation generated by xenon excimer lamps has been investigated [6,8,43]. VUV irradiation converts PHPS into silicon oxide networks through direct photochemical bond cleavage and subsequent oxidation by atmospheric oxygen species.

**Figure 4:**
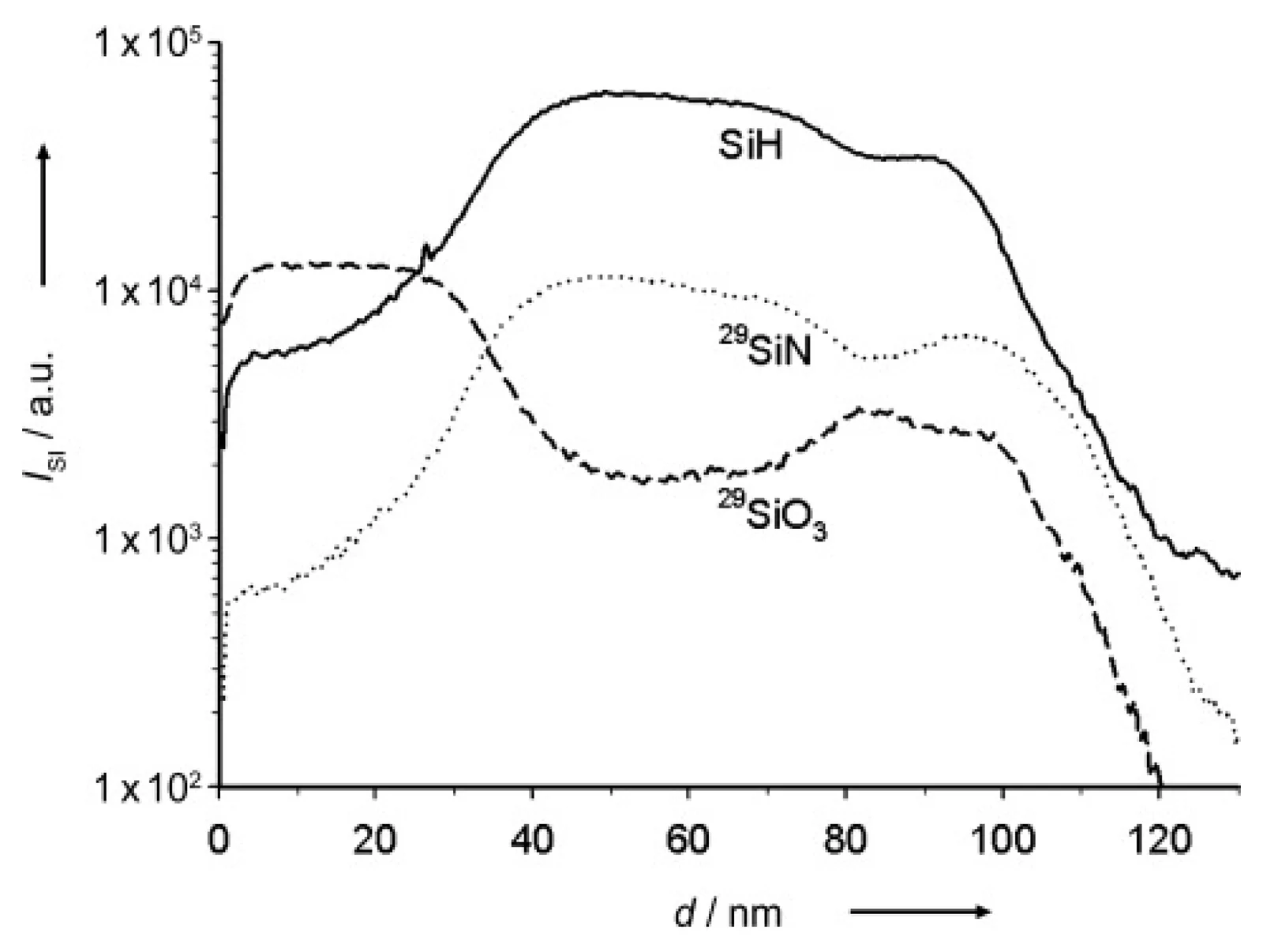
Depth-resolved TOF-SIMS profiles of spin-coated PHPS films deposited on Si wafers following VUV irradiation using a Xe_2_* excimer lamp at a dose of 2.1 J cm^−2^. I_SI_: the secondary ion current intensity, d: Penetration depth. Reprinted with permission from Ref. [8]. Copyright 2007, WILEY-VCH Verlag GmbH & Co. KGaA, Weinheim, Germany.

As discussed in Section 3.2.1, VUV-assisted curing exhibits substantially lower volumetric shrinkage than thermal curing, making it advantageous for the fabrication of dense films with improved dimensional stability [6]. However, the effective penetration depth of 172 nm photons is limited to approximately 200 nm. Time-of-flight secondary ion mass spectrometry (TOF-SIMS) depth profiling revealed that PHPS is extensively converted into SiO_x_ in the near-surface region (0–30 nm), whereas the degree of conversion decreases in the underlying layers, resulting in a higher concentration of Si–N species at greater depths (Figure 4) [8]. Consequently, in films thicker than the effective penetration depth, the upper region is converted predominantly into silica, while residual nitrogen-containing species remain in the lower region, leading to the formation of a compositional gradient through the film thickness [26].

#### 3.2.3. Catalytic Curing

Catalytic curing utilizes the intrinsic chemical reactivity of PHPS to induce conversion at room temperature or under very mild thermal conditions.

##### Ammonia (NH_3_) and Amine Vapor Exposure

When PHPS films are exposed to ammonia vapor generated from aqueous ammonia at room temperature, base-catalyzed hydrolysis occurs [12]. This treatment reduces the nitrogen content from 33.6% to below 0.5% and increases the pencil hardness from less than 6B to greater than 9H. In addition, the resulting films exhibit denser and more compact structures than those prepared by conventional alkoxide-based sol–gel processes using precursors such as tetraethyl orthosilicate (TEOS) [16]. Depth-profile analyses revealed no significant gradients in nitrogen or oxygen concentration throughout the film thickness [11].

##### Hydrogen Peroxide (H_2_O_2_)

PHPS was successfully converted using H_2_O_2_ without the need for UV irradiation, achieving curing within only 10 min [49]. Conversion to SiO_x_ was also observed when NH_4_OH, another source of hydroxyl species, was employed at room temperature. Interestingly, whereas thermally converted films typically exhibit a shrinkage of approximately 19%, H_2_O_2_-cured films showed a slight expansion of about 2%. Based on IR spectroscopic analyses, this unusual expansion behavior was attributed to the formation of cagelike Si–O network structures within the film.

##### 3-Aminopropyltriethoxysilane (APTES)

The incorporation of catalysts such as APTES into PHPS solutions has also been investigated to accelerate the curing process. Complete curing was achieved within only 10 min under low-temperature and high-humidity conditions, while the volumetric shrinkage was suppressed to approximately 0.86%. Because film shrinkage is a potential source of defects, minimizing volumetric shrinkage is advantageous for reducing defect formation. In addition, it helps alleviate coating stress arising from volume mismatch between the coating layer and the substrate [3].

#### 3.2.4. Combined VUV–Thermal Curing

Channa and co-workers reported that one of the fastest conversion methods involves deep-UV irradiation of PHPS combined with heating at 100 °C. This approach yielded a water vapor transmission rate (WVTR) of approximately 10^−3^ g m^−2^ day^−1^ and an oxygen transmission rate (OTR) of less than 10^−1^ cm^3^ m^−2^ day^−1^ bar^−1^. Owing to its rapid low-temperature conversion and excellent barrier performance, this process was proposed as a promising encapsulation strategy for organic solar cells and related optoelectronic devices. The authors further demonstrated that the conversion rate could be enhanced by reducing the distance between the deep-UV light source and the sample to approximately 5 mm [15].

### 3.3. Preparation of SiON-Based Materials

The preparation of silicon oxynitride (SiO_x_N_y_) films from PHPS follows conversion routes similar to those employed for SiN_x_ and SiO_x_ materials. In general, both thermal curing and UV-assisted curing methods have been utilized to induce the conversion of PHPS into SiON-based networks.

#### 3.3.1. Low-Temperature Thermal Curing

The most widely employed approach for the preparation of PHPS-derived SiO_x_N_y_ materials is thermal curing under ambient air or controlled humidity conditions. Unlike the high-temperature pyrolytic conversion used for SiN_x_ preparation, SiO_x_N_y_ formation from PHPS is typically achieved through low-temperature thermal curing, where partial oxidation and hydrolysis occur while a portion of the Si–N network is retained.

**Figure 5:**
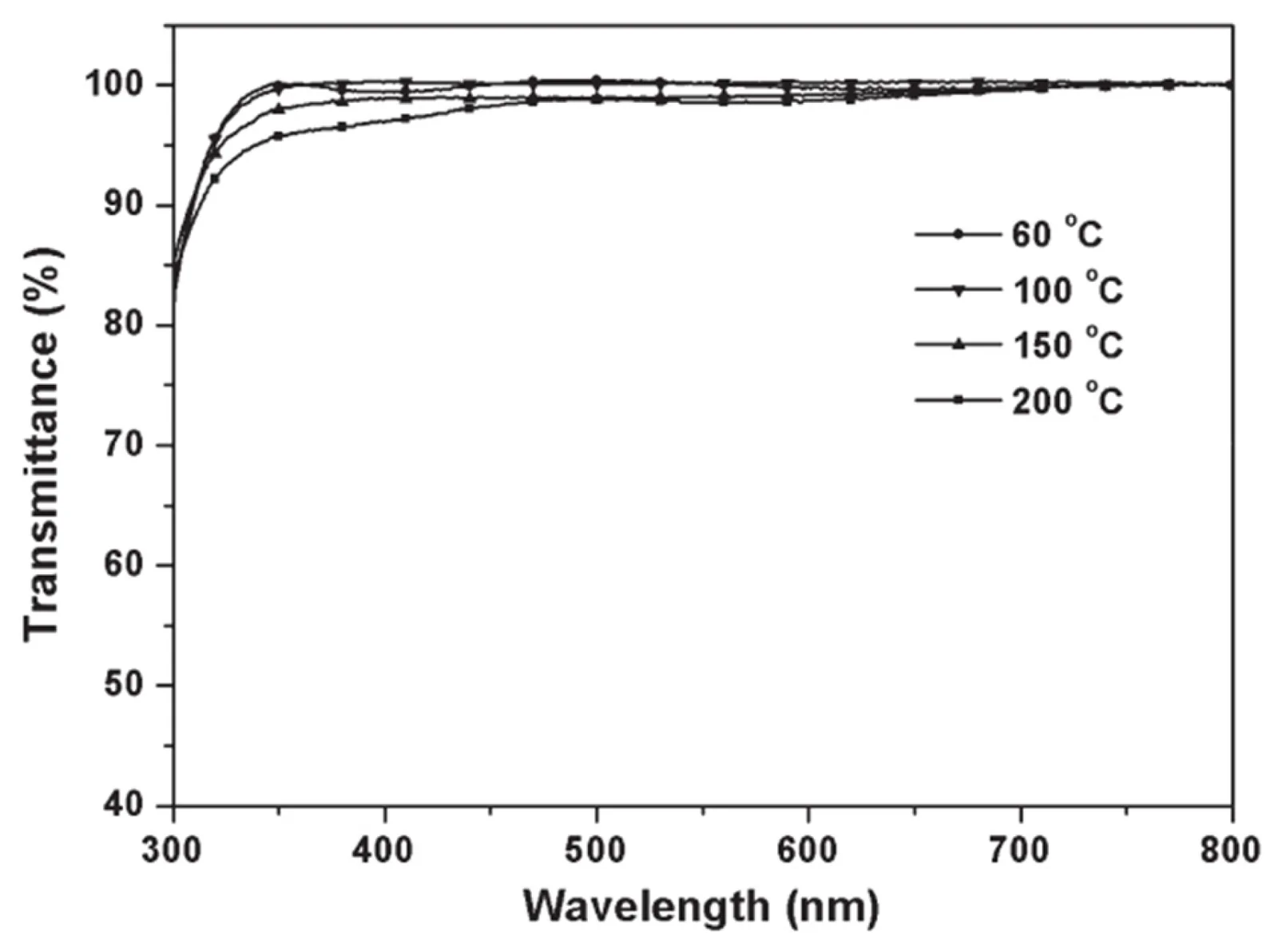
Transmittance of PHPS-derived coatings on glass substrates after annealing at different temperatures. Reprinted with permission from Ref. [14]. Copyright 2015, Society of Chemical Industry.

For example, hydrophobic SiO_x_N_y_ coatings were prepared by heating PHPS at temperatures ranging from 60 to 200 °C for 2 h. The hardness of the resulting coatings increased with increasing curing temperature, while maintaining a visible-light transparency exceeding 90% (Figure 5) [14]. In another study, PHPS was thermally cured at 70 °C for various durations to produce SiO_x_N_y_ solids. X-ray photoelectron spectroscopy (XPS) analysis revealed a decrease in the nitrogen content from 22.71% to 3.38%, accompanied by a corresponding increase in oxygen content, indicating the progressive conversion of PHPS into SiO_x_N_y_ during thermal treatment [50].

Thermally cured SiO_x_N_y_ layers have also been investigated for semiconductor packaging applications. To suppress the delamination of Cu/epoxy molding compound (EMC) interfaces, PHPS was sequentially heated at 120 °C for 60 min and subsequently at 150 °C for an additional 60 min to form a SiO_x_N_y_ interfacial layer [51]. In addition, PHPS-derived SiO_x_N_y_ coatings have been employed for the surface modification of Si_3_N_4_ fibers, where thermal treatment was conducted at approximately 100 °C for several hours [52]. A related study further promoted the hydrolysis-driven conversion process by maintaining a high relative humidity of 80% during curing at 100 °C for 4 h [53].

#### 3.3.2. UV-Induced Curing

In addition to low-temperature thermal curing, UV-induced curing has also been investigated for the formation of PHPS-derived SiO_x_N_y_ coatings. In a representative study, PHPS-derived SiO_x_N_y_ thin films were prepared using two independent low-temperature curing methods: either by thermal annealing at 180 °C for 60 min or by UV-induced curing using lamps emitting at 185 and 254 nm for 40 min, to compare the resulting chemical compositions and properties [54,55]. As discussed in Section 3.2.2, unlike thermal curing, UV-induced curing produces a depth-dependent compositional gradient in which the surface region is rapidly converted into SiO_2_ with little or no residual nitrogen, whereas the nitrogen content gradually increases toward the film interior. In contrast, thermal curing generally produces films with a relatively uniform composition throughout the film thickness.

#### 3.3.3. Comparison of Curing Processes and Chemical Transformations

The conversion of PHPS into SiO_x_N_y_ proceeds through reactions with moisture and oxygen in the atmosphere, following pathways similar to those involved in the formation of SiN_x_ and SiO_x_ materials. Under humid conditions, the Si–N and Si–H bonds in PHPS undergo hydrolysis to form Si–OH groups, which subsequently condense to generate Si–O–Si networks. During this conversion process, ammonia and hydrogen are released as gaseous byproducts. These chemical transformations have been confirmed by FTIR analyses, which show the gradual attenuation or disappearance of the characteristic Si–H (approximately 2160–2170 cm^−1^) and N–H (approximately 3380 cm^−1^) absorption bands during curing [14,49,51,53]. Shmagina and co-workers reported that UV-cured films exhibit lower nitrogen contents than thermally cured films and consequently show refractive indices closer to that of silica (approximately 1.46). They further observed that thermally cured films immediately after processing possess relatively high ductility but limited adhesion. However, after an aging period of approximately one week, substantial improvements in hardness and adhesion were observed. This behavior was attributed to the gradual replacement of nitrogen by oxygen within the film, resulting in material characteristics comparable to those of UV-cured coatings [55].

## 4. Conversion Mechanisms of PHPS into Silicon-Based Networks

This section discusses the conversion mechanisms of PHPS in two primary parts. Section 4.1 addresses atmosphere-dependent thermal and hydrolytic conversion under various environments, including humid, NH_3_/H_2_O, O_2_/H_2_O, and anhydrous NH_3_ conditions [1,2,7,11,12,56]. Section 4.2 then examines catalyst-assisted, radical-mediated, and photoinduced conversion pathways [3,6,8,17,38,39,49,57,58,59]. Together, these discussions clarify how reaction atmosphere and external activation routes modulate the conversion kinetics, network composition, and structural evolution of PHPS-derived silicon-based inorganic networks.

### 4.1. Atmosphere-Dependent Thermal and Hydrolytic Conversion of PHPS

PHPS conversion is an atmosphere-dependent structural reorganization process in which the evolving network ultimately forms oxide, oxynitride, or nitride structures [1,2,7,56]. During the early stage of conversion, PHPS releases H_2_ through dehydrogenative coupling between Si–H and N–H groups, accompanied by the formation of new Si–N bonds and the development of a crosslinked inorganic structure. This crosslinked structure subsequently evolves into the final silicon-based structure according to the curing atmosphere.

In the presence of moisture or NH_3_/H_2_O environments, Si–O–Si-based networks develop through the hydrolysis of the PHPS network, particularly Si–N bonds, followed by condensation reactions [11,12]. In contrast, under oxygen-deficient conditions or nitrogen-containing atmospheres such as anhydrous NH_3_, Si–N-based bonding environments may be retained or further reinforced, favoring the formation of SiO_x_Nᵧ- or SiN_x_-based networks [1,2,7,56]. Therefore, the thermal and hydrolytic conversion of PHPS should be regarded not as a single oxidation reaction but as a multifaceted process in which hydrolysis, oxidation, nitrogen removal, dehydrogenation, nitridation, and structure rearrangement are competitively coupled depending on the reaction atmosphere and temperature [1,2,7,11,12,56].

Interestingly, Hou et al. used ReaxFF molecular dynamics simulations to predict that PHPS conversion into SiO_x_/SiO_x_Nᵧ proceeds through competing H_2_O-driven and O_2_-driven pathways [2]. H_2_O preferentially promotes hydrolytic cleavage of the Si–N backbone because the calculated barrier for Si–N hydrolysis is approximately 10 kcal mol^−1^ lower than that for Si–H hydrolysis, leading to nitrogen removal mainly as NH_3_. In contrast, O_2_ more strongly promotes Si–H oxidation, rapid Si–O bond formation, and the development of a SiO_x_-rich surface layer. However, in condensed-phase film simulations, this surface layer acts as a diffusion barrier: under an O_2_ atmosphere, nitrogen removal remained near 10%, the O/Si ratio reached only about 0.8, and only about 50% of O_2_ molecules could enter the film after the surface oxide layer had developed. Thus, O_2_-rich conversion tends to produce a depth-dependent Si–O bonding gradient, whereas H_2_O-rich conditions form a less compact SiO_x_ network with a weaker diffusion-limiting effect [2].

PHPS conversion has been shown to proceed rapidly even at low temperatures when hydrolysis is accelerated under basic humid conditions, as demonstrated by studies involving exposure to vapor from aqueous NH_3_ or NH_3_/H_2_O-containing atmospheres [11,12]. In particular, exposure to vapor from aqueous ammonia at room temperature provides a representative low-temperature pathway for promoting PHPS hydrolysis and condensation, enabling transformation into a Si–O–Si-based structure without high-temperature thermal treatment. In this process, H_2_O and NH_3_ molecules are considered to adsorb on the PHPS film surface, where OH^−^ species promote base-catalyzed hydrolysis through nucleophilic attack on Si centers [12]. This reaction leads to the consumption of Si–N-containing structures and Si–H/N–H bonds, followed by the formation and rapid condensation of Si–OH or other oxygen-containing intermediates into Si–O–Si linkages [11,12]. Similarly, PHPS conversion in NH_3_/H_2_O-based basic humid atmospheres is understood to proceed through a base-catalyzed hydrolysis–condensation mechanism. Nakajima et al. reported that the absence of a significant oxygen or nitrogen compositional gradient across approximately 500 nm-thick films indicates that the conversion proceeds primarily as a reaction-limited rather than diffusion-limited process [11].

However, atmosphere-regulated PHPS conversion does not always culminate in the complete formation of SiO_2_. Under low-temperature or humid curing conditions, Si–N hydrolysis and nitrogen removal may remain incomplete, allowing residual Si–N-containing environments to persist and SiO_x_Nᵧ or other nitrogen-containing networks to form rather than fully oxidized SiO_2_ [14,45,51]. ReaxFF molecular dynamics simulations further indicate that the Si–O–N composition of PHPS-derived networks can be tuned by controlling the H_2_O/O_2_ ratio and overall oxygen availability during thermal conversion [2]. Consequently, SiO_x_Nᵧ should be understood not merely as a transient composition toward complete oxidation, but also as a meaningful intermediate or target network composition, as experimentally demonstrated in low-temperature thermally converted PHPS coatings [14,51]. Whether this composition is retained or further converted depends on the H_2_O/O_2_ atmosphere and oxygen availability [2], as well as the curing temperature, moisture level, and treatment duration [14,45].

**Scheme 1:**
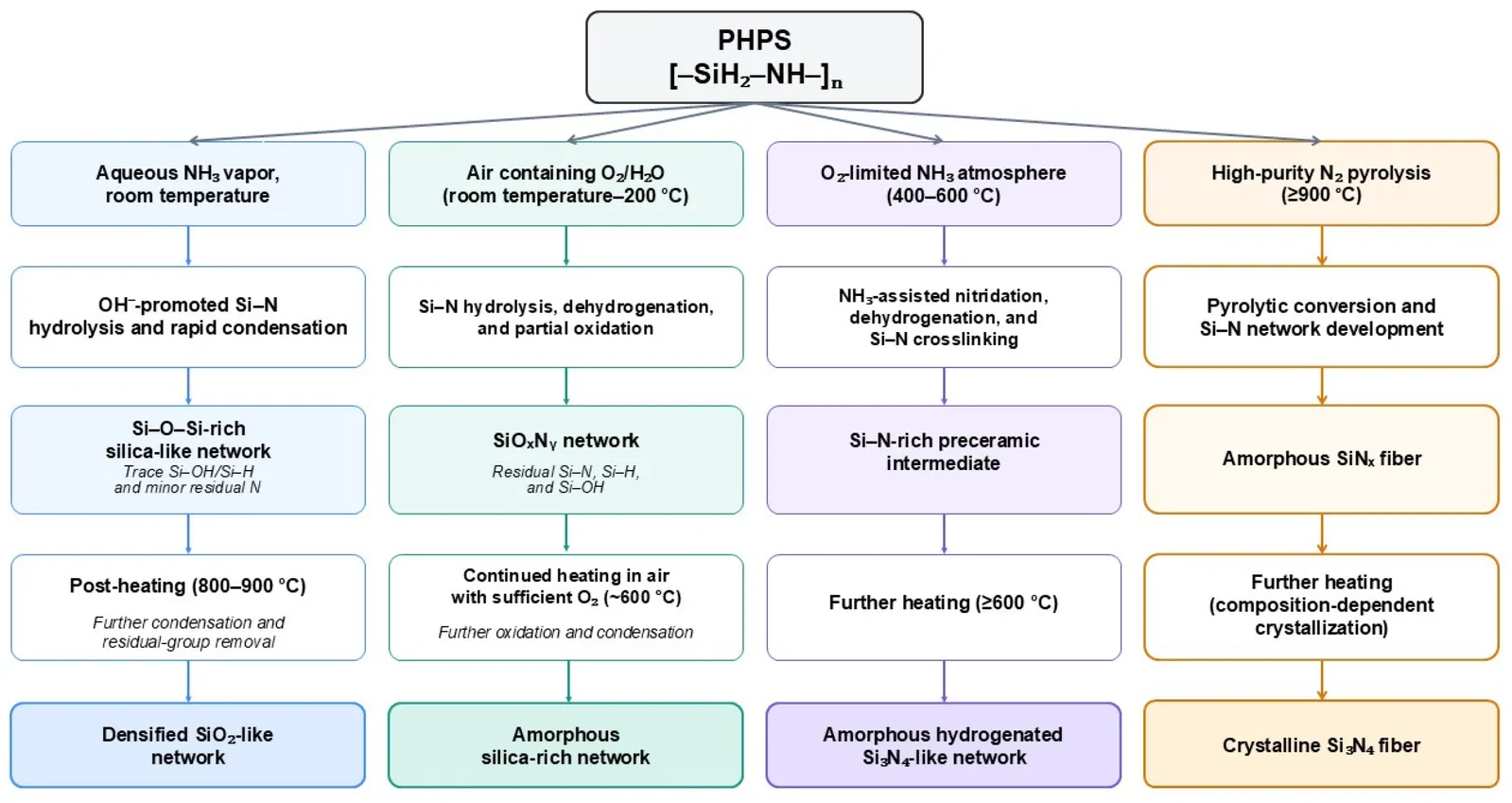
Representative atmosphere-dependent thermal and hydrolytic conversion pathways of PHPS. The arrows indicate the sequential progression of PHPS conversion under each set of conditions.

In contrast, when oxygen supply is restricted and heat treatment is performed under an anhydrous NH_3_ atmosphere, PHPS conversion favors nitridation over the formation of Si–O-based networks [7,56]. Funayama et al. examined the thermal decomposition of PHPS in flowing anhydrous ammonia up to 1000 °C and divided the conversion into three temperature regimes. At temperatures below 400 °C, the main process is the removal of residual solvent, with little apparent rearrangement of the polymer framework. Between 400 and 600 °C, NH_3_ participates in the transformation of Si–H-containing environments into Si–N-rich bonding structures, accompanied by an increase in nitrogen content [7]. This stage represents an intermediate state between the polymeric precursor and the ceramic product, characterized by the development of three-dimensional Si–N connectivity. Consistently, ^29^Si nuclear magnetic resonance (NMR), XPS, and X-ray diffraction analyses indicate progressive growth of the Si–N network and a structural shift toward an amorphous Si_3_N_4_-like configuration. At higher temperatures, from 600 to 1000 °C, the overall structural evolution becomes less pronounced, and the material approaches a hydrogen-containing amorphous silicon nitride composition. Therefore, heat treatment under anhydrous NH_3_ is best described as a nitridation–ceramization pathway in which the Si–H/Si–N-based PHPS framework is progressively reorganized into a three-dimensional Si–N network with increasing nitrogen incorporation [7]. In contrast to the studies described above, PHPS was thermally decomposed under a high-purity nitrogen atmosphere, with no exposure to air and in the absence of catalysts or other additives, to form a Si–N network [40]. Heating PHPS above 900 °C resulted in the formation of amorphous silicon nitride ceramic fibers, which remained predominantly amorphous with increasing temperature and exhibited high mechanical strength near 1400 °C. Above 1400 °C, crystallization of α-Si_3_N_4_ began, accompanied by a decrease in weight attributed to the removal of oxygen-containing species that had been introduced by unavoidable exposure to moisture. The representative atmosphere-dependent thermal and hydrolytic conversion pathways of PHPS are outlined in Scheme 1.

### 4.2. Catalytic, Radical-Mediated, and Photoinduced Conversion of PHPS

Beyond thermal and hydrolytic conversion, PHPS can also be transformed through various low-temperature or non-thermal activation routes, including catalytic activation, radical-mediated oxidation, and UV/VUV irradiation [3,6,17,38,39,49,57,58,59]. These external activation routes facilitate or redirect PHPS conversion by inducing Si–H and Si–N bond cleavage, followed by hydrolysis or oxidation, oxygen incorporation, condensation reactions, and network densification, depending on the activation mode and reaction environment.

Low-temperature conversion of PHPS using APTES represents a catalytic hydrolysis–condensation pathway in which the amino groups of APTES facilitate the dissociation of water molecules, thereby catalyzing the hydrolysis of Si–H and Si–N bonds to generate Si–OH intermediates that subsequently condense into Si–O–Si linkages [3]. This APTES-assisted conversion is strongly dependent on temperature and humidity, which enhance water diffusion into the PHPS coating and favor Si–H/Si–N hydrolysis and Si–O bond formation. Notably, PHPS containing 5 wt% APTES was converted within 10 min at 80 °C and 90% relative humidity, with approximately 95% of Si–H bonds and 84% of Si–N bonds being consumed. A related amine-based catalytic route was reported by Je et al. using 2-dimethylamino-1-propanol (DMAPO) as an additive in a PHPS solution. Under humid conditions, DMAPO acted as an alkaline catalyst that facilitated Si–H/Si–N hydrolysis, silanol formation, and subsequent condensation, enabling the formation of a PHPS-derived SiO_2_ network at a low temperature of 180 °C [57].

**Scheme 2:**
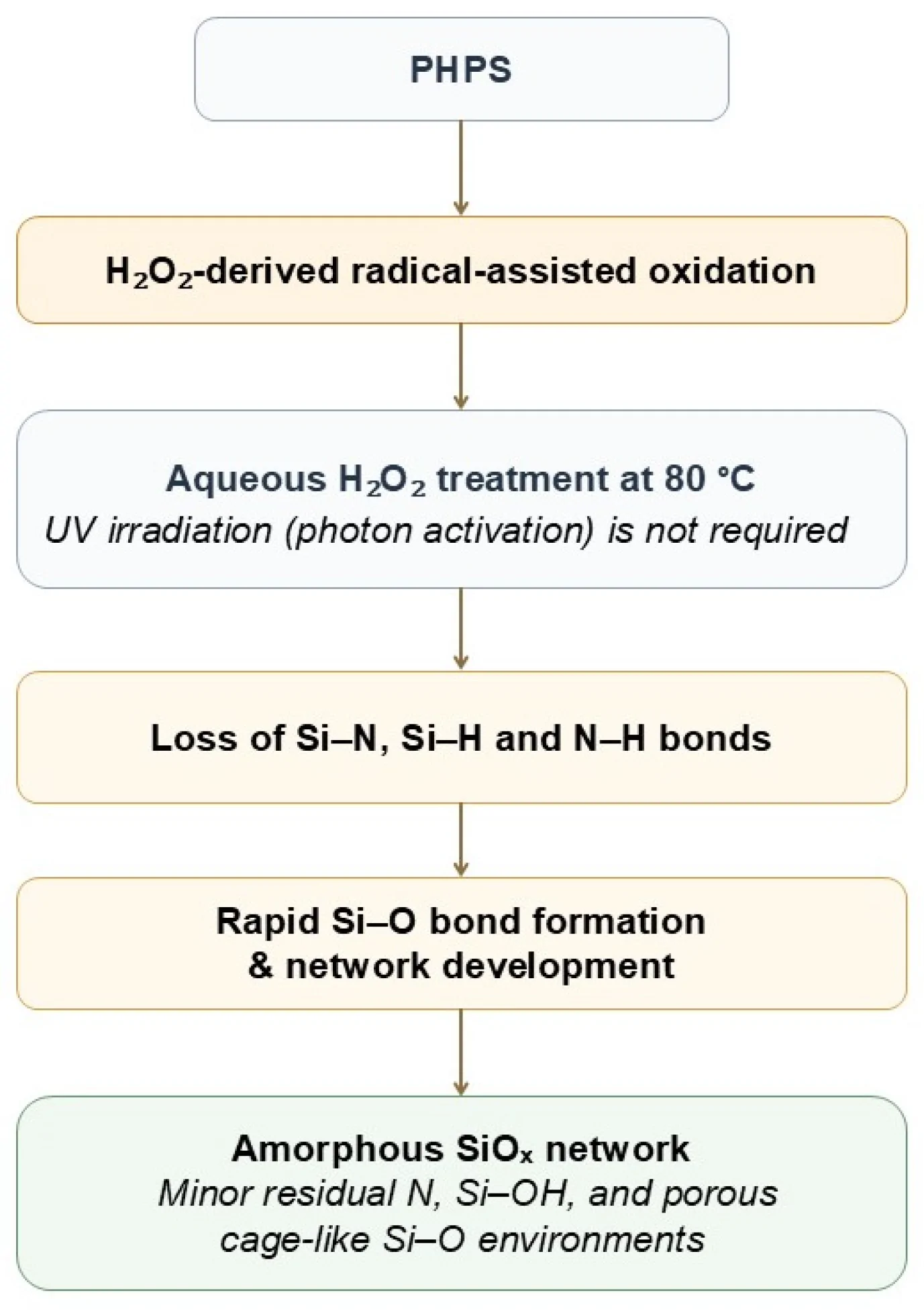
H_2_O_2_-Assisted Oxidative Conversion of PHPS.

Unlike catalyst-assisted hydrolysis–condensation pathways, peroxide- or radical-assisted conversion relies more strongly on oxidative activation. Jung et al. showed that dipping PHPS coatings in an aqueous H_2_O_2_ solution at 80 °C for 10 min effectively converted PHPS into a SiO_x_ network [49]. Consistently, the Si–N, Si–H, and N–H absorption bands disappeared within 10 min during H_2_O_2_ solution curing, regardless of UV irradiation, while Si–O absorption increased markedly. The authors propose that H_2_O_2_ can generate hydroxyl radicals (•OH) even without photon activation (Scheme 2).

A different photochemical strategy was reported by Seul and coworkers [58]. Deep-ultraviolet (DUV)-assisted low-temperature conversion was achieved by incorporating vinyltriethoxysilane (VTES) and DMAPO into a PHPS precursor solution, followed by DUV irradiation under an N_2_ atmosphere. In this system, VTES promotes the formation of Si–O networks by reducing the hydrolysis temperature of PHPS and facilitating the replacement of Si–N bonds with Si–O bonds, leading to the formation of Si–O–Si and Si–O–R linkages. During DUV annealing, photons with energies of approximately 4.9 and 6.5 eV are sufficient to cleave Si–N and Si–H bonds in PHPS, whose reported bond energies are 4.87 and 3.6 eV, respectively. Under these conditions, additive-assisted Si–O formation and DUV-induced photochemical reactions promote Si–O network growth, polycondensation, impurity removal, and film densification, yielding a densified SiO_2_-dominant network with reduced C, N, and OH contents but residual SiO_x_Nᵧ environments [58].

Prager et al. proposed that VUV-induced conversion of PHPS into SiO_x_ is initiated not by immediate Si–O–Si network formation but by photochemical Si–N bond cleavage [8]. Quantum-chemical calculations showed that VUV excitation induces intramolecular charge redistribution from nitrogen toward silicon, thereby weakening the Si–N bond and enabling its scission. Kinetic FTIR analysis further demonstrated that the decay of Si–H-related absorption precedes the growth of Si–O–Si bands, indicating that bond cleavage occurs before oxidative network formation. The resulting Si-centered radical species subsequently react with oxygen to form the final SiO_x_ network [8]. Naganuma et al. demonstrated that oxygen concentration strongly influences the VUV-assisted conversion pathway [6]. At 21% oxygen, PHPS was converted into silica throughout the film thickness, whereas under oxygen-deficient conditions, silica formation was largely confined to the near-surface region and silicon oxynitride remained within the film. Notably, silica formation was still observed at extended irradiation distances where direct VUV irradiation of the PHPS surface was strongly attenuated, indicating that oxygen-derived reactive oxygen species (ROS), including O(^1^D), O_3_, and ^1^O_2_, can sustain surface-initiated oxidation. These results suggest that VUV-assisted PHPS conversion proceeds through a coupled process involving both direct photochemical bond scission and ROS-mediated oxidation [6]. Kobayashi et al., however, demonstrated that ROS-mediated oxidation is not always the dominant pathway [59]. They reported the lowest WVTR for PHPS-derived silica coatings under conditions that maximized the transmittance of 172 nm VUV light to the coating surface, namely, an oxygen concentration below 5% and an irradiation distance of 2.6 mm. Under these conditions, direct VUV-induced Si–N bond cleavage, rather than oxidation mediated by O_3_ or O(^1^D), was proposed to play the dominant role in producing dense gas-barrier silica coatings [59]. Morlier et al. further demonstrated that UV/VUV-cured PHPS films develop a depth-dependent compositional gradient because oxygen diffusion becomes progressively limited during conversion [17]. FTIR and TOF-SIMS analyses revealed a SiO_x_-rich surface layer, a SiO_x_Nᵧ intermediate region, and a hydrogenated silicon nitride-like deeper region. In the oxygen-deficient interior, VUV irradiation promotes condensation between Si–H and N–H groups, generating additional Si–N linkages through an oxygen-independent transformation. Collectively, these studies indicate that VUV-assisted PHPS conversion is a condition-dependent photochemical process in which the relative contributions of direct bond cleavage, ROS-mediated oxidation, oxygen-diffusion limitation, and Si–N network reconstruction vary with oxygen concentration, irradiation distance, irradiation dose (or exposure time), and film thickness [6,8,17,59].

In contrast to VUV-induced SiO_x_ conversion in oxygen-containing atmospheres, VUV irradiation under an inert atmosphere provides a photochemical route for converting and densifying PHPS films into SiN-based networks [39]. Sasaki et al. investigated the densification behavior of VUV-converted PHPS-derived silicon nitride (PDSN) films under 172 nm VUV irradiation in an N_2_ atmosphere. They showed that initial densification occurs through the rapid formation of Si–N bonds with concomitant H_2_ release, triggered by photochemical reactions of Si–H and N–H bonds. However, after prolonged irradiation, the film refractive index continued to increase after the formation of new Si–N bonds had plateaued, indicating that densification cannot be explained solely by an increase in the number of Si–N bonds. This later-stage densification was attributed to VUV-induced repeated cleavage and reformation of Si–N bonds, followed by atomic rearrangement within the Si–N network. As a result, free-volume reduction in the Si–N network proceeds more slowly than the initial Si–H/N–H photochemical reaction and acts as the rate-determining process for densification [39].

**Figure 6:**
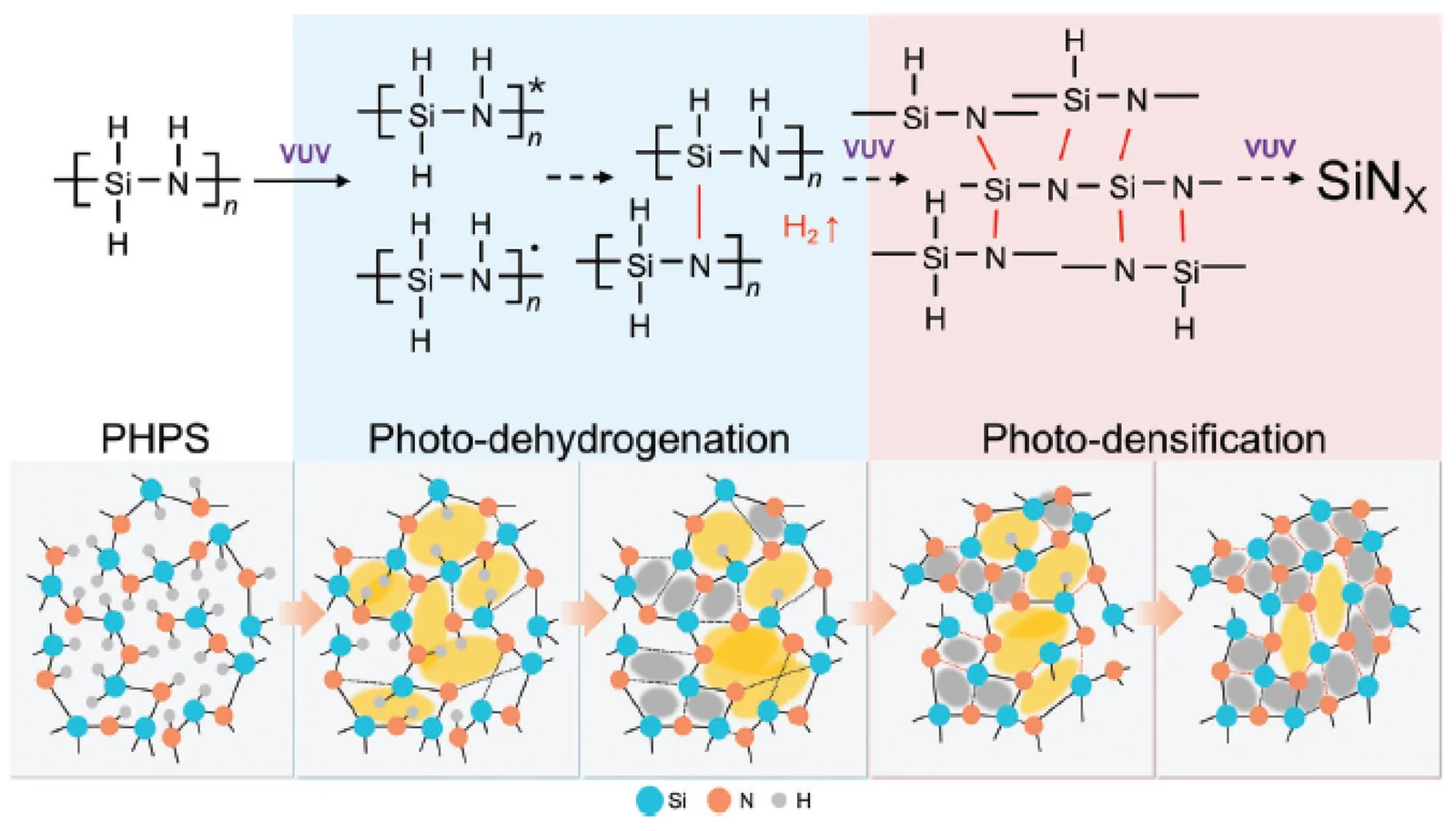
Schematic illustration of the photo-dehydrogenation and photo-densification processes during VUV conversion of PHPS. In the upper chemical scheme, the solid black arrow denotes the initial VUV activation of PHPS, whereas the dashed black arrows indicate the proposed subsequent reaction steps. The asterisk (*) and dot (•) denote intermediate and radical species, respectively, and the red lines represent newly formed or rearranged Si–N bonds. Reprinted with permission from Ref. [38]. Copyright 2025, The Author(s).

**Scheme 3:**
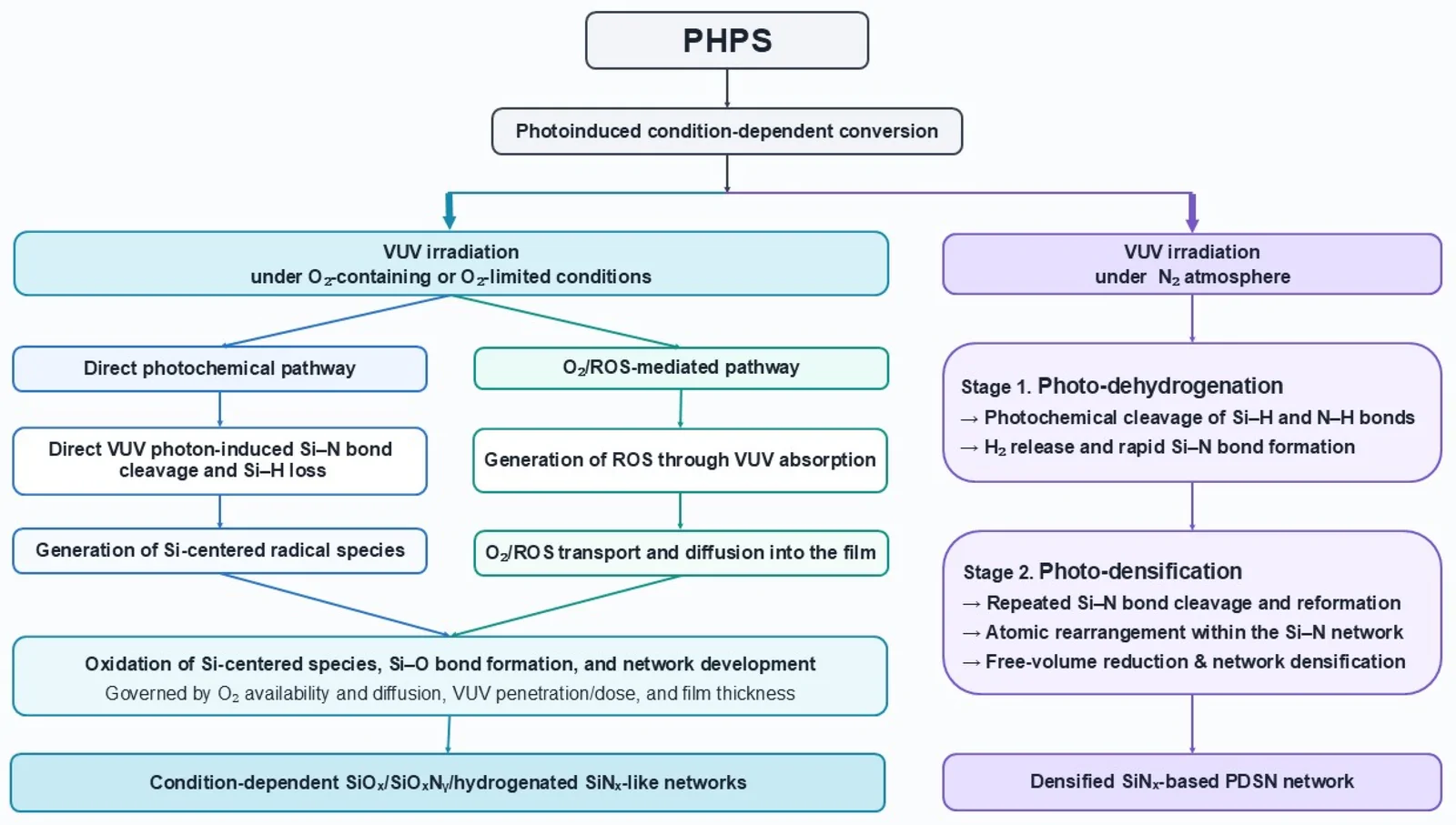
Representative photoinduced conversion pathways of PHPS.

Recently, high-power VUV processing has been implemented for PDSN film formation, significantly reducing single-layer irradiation times to a range of sub-10 s to approximately 20 s, depending on the irradiation dose [38]. Song et al. distinguished the 172 nm VUV-induced conversion of PHPS into photo-dehydrogenation and subsequent photo-densification (Figure 6). The initial photo-dehydrogenation stage, involving Si–H/N–H bond cleavage and Si–N bond formation, proceeded mainly as a dose-dependent, intensity-independent single-photon process. By contrast, photo-densification showed strong VUV-intensity dependence; above approximately 290 mW cm^−2^, repeated Si–N bond cleavage/reformation and network rearrangement facilitated free-volume reduction, enabling rapid densification of PHPS-derived SiN_x_ films. Scheme 3 delineates the atmosphere- and condition-dependent VUV-induced PHPS conversion pathways.

Overall, catalyst-, radical-, and photo-assisted routes broaden PHPS conversion beyond conventional thermal or hydrolytic pathways by enabling low-temperature Si–H/Si–N bond cleavage, oxygen incorporation, condensation, and network densification [3,6,8,17,38,39,49,57,58,59]. In oxygen-containing atmospheres, VUV conversion reflects a balance between photon-induced bond scission and ROS-mediated oxidation, whereas VUV irradiation under inert atmospheres favors the formation of PHPS-derived SiN_x_ networks through photo-dehydrogenation and subsequent photo-densification [6,8,17,38,39,59].

## 5. Properties of PHPS-Derived Silicon-Based Networks

### 5.1. Composition and Structural Characteristics

PHPS-derived silicon-based networks do not necessarily converge into a single homogeneous amorphous silica phase. Instead, their composition, local bonding environments, and network structures evolve differently depending on the conversion atmosphere. ReaxFF molecular dynamics simulations demonstrated that O_2_ and H_2_O induce distinct atomistic conversion pathways, leading to atmosphere-dependent differences in Si–O coordination and the prevalence of fourfold N-coordinated Si sites. In the O_2_-containing simulation, the time-dependent evolution of Si–O–Si ring structures was also observed. Accordingly, PHPS-derived films are more appropriately regarded as atmosphere-dependent silicon-based networks containing diverse Si–O and Si–N bonding environments rather than as uniform amorphous silica [2].

Under basic humid conditions, PHPS is converted at room temperature into films with a nearly stoichiometric silica composition; however, such films should not be assumed to possess the same structural state as silica glass. Exposure to vapor from aqueous ammonia yielded PHPS-derived silica films with O/Si molar ratios close to 2 and refractive indices comparable to that of silica glass [11]. Nevertheless, trace Si–OH and Si–H groups remained, and UV absorption absent from silica glass was still observed. Thus, even when the O/Si ratio approaches the stoichiometric value of silica and the refractive index becomes silica-glass-like, the resulting film cannot be regarded as structurally equivalent to fully dense silica glass.

Vapor from aqueous ammonia treatment at room temperature also caused concurrent decreases in nitrogen content, refractive index, and water contact angle, indicating substantial progress in PHPS-to-silica conversion [12]. The exposure-treated films nevertheless remained distinguishable from pure, dense silica glass. Subsequent firing at 900 °C further reduced the nitrogen content from 0.53 to 0.01 atomic%, while the refractive index decreased from 1.48 to 1.45 and the water contact angle from 67° to 29°, bringing these properties closer to those of silica glass [12]. These observations further demonstrate that elemental composition alone is insufficient to describe the structural state of PHPS-derived silica [11,12].

In contrast, APTES-catalyzed conversion at 80 °C and 90% relative humidity rapidly transformed PHPS into a silica-dominant coating within 10 min [3]. FTIR analysis showed marked decreases in the Si–H and Si–N signals together with progressive Si–O bond formation. The resulting coating still had an O/Si ratio of 1.74 and contained 1 atomic% residual nitrogen. Solid-state ^29^Si NMR further revealed a mixed bonding environment dominated by SiNO_3_/SiO_3_OH and SiO_4_ units, with a smaller contribution from HSiO_3_/SiN_2_O_2_-type environments. Its density was also lower than that of amorphous silica [3]. Thus, consistent with the previous examples, although low-temperature conversion produced a relatively homogeneous silica-dominant coating, the resulting structure should not be regarded as equivalent to fully dense pure silica.

Within this structural continuum, the persistence of nitrogen-containing environments should not be interpreted solely as evidence of incomplete conversion. PHPS-derived silicon oxynitride may instead represent a chemically and functionally meaningful intermediate network state prior to complete transformation into SiO_2_ [14].

VUV-induced conversion produced oxygen-concentration-dependent variations in the composition of PHPS-derived films. As discussed in Section 3.2.2 and Section 4.2, the limited penetration depth of 172 nm VUV light leads to depth-dependent conversion, giving rise to compositional and structural gradients across UV/VUV-cured PHPS films [8]. Consistent with this behavior, PHPS films thicker than 30 nm did not fully convert to homogeneous silica under oxygen-depleted conditions [17]. Instead, they formed a graded architecture comprising a 30–50 nm silicon oxide surface layer, an approximately 200 nm silicon oxynitride intermediate layer with a decreasing O/N ratio toward the interior, and a deeper hydrogenated silicon nitride layer. After VUV irradiation and subsequent aging, PHPS-related N–H and Si–H bands remained detectable, while XPS depth profiles of selected coatings showed higher nitrogen concentrations in the film interior [59]. Collectively, these results indicate that nominally single-layer UV/VUV-cured PHPS coatings may contain graded SiO_x_-, SiO_x_Nᵧ-, and Si–N-rich regions whose depth distribution may influence barrier performance, rather than forming a fully homogeneous silica layer [8,17,59].

PHPS-derived structures are not restricted to oxide or oxynitride states. Combined in situ XPS and X-ray absorption near-edge structure measurements, together with FTIR and temperature-programmed desorption analyses, showed that dehydrogenation became pronounced between 400 and 600 °C during heat treatment under vacuum and low-pressure NH_3_ atmospheres [56]. The resulting dangling bonds provided reactive sites for nitrogen incorporation from NH_3_, promoting the formation of an extended Si–N network based on tetrahedrally coordinated Si centers. With sufficient NH_3_, even Si–O bonds introduced by residual moisture were replaced by Si–N bonds, driving the network toward a Si_3_N_4_-like structure. This nitridation pathway is consistent with pyrolytic conversion results showing that PHPS formed an amorphous covalent Si–N ceramic at approximately 700 °C and subsequently underwent Si_3_N_4_ crystallization at 1260 °C [1]. These results indicate that nitrogen-rich PHPS-derived networks are not merely transient intermediate states but may serve as precursors to crystalline Si_3_N_4_-containing ceramics [1,56].

Collectively, PHPS conversion gives rise to a broad structural continuum rather than a single network composition. Depending on the conversion atmosphere, oxygen availability, temperature, and activation method, the resulting structures range from SiO_x_-rich networks through SiO_x_Nᵧ intermediate compositions to nitrogen-rich SiN_x_ networks [2,3,6,8,11,12,14,17,59]. Under NH_3_-assisted thermal conversion, these nitrogen-rich networks may further evolve into Si_3_N_4_-like structures and eventually crystallize into Si_3_N_4_-containing ceramics during subsequent high-temperature treatment [1,56]. Likewise, VUV irradiation under N_2_ produces densified PDSN networks through photoinduced nitridation [39].

### 5.2. Refractive Index and Transparency of PHPS-Derived Films

The refractive index and optical transmittance of PHPS-derived films are governed by the conversion pathway, the resulting O/N composition, residual Si–N and Si–H bonding environments, and the degree of network densification [39,49,60]. In particular, the refractive index primarily reflects changes in network composition and density, whereas optical transmittance is additionally influenced by film thickness, interference effects, substrate optical properties, and surface morphology [14,19,43,60].

As discussed in Section 5.1, exposure of PHPS films to vapor from aqueous ammonia at room temperature reduced the refractive index to a value close to that of silica glass. However, despite this silica-glass-like refractive index, the converted films remained structurally distinct from dense silica glass because residual nitrogen-containing species and an incompletely condensed network were still present [12].

A similar trend was observed for a low-temperature liquid-phase conversion route. Treatment of PHPS films in a 30% aqueous H_2_O_2_ solution at 80 °C converted them into SiO_x_ films, with refractive indices of approximately 1.45–1.46 after 10–60 min, close to that of pure fused silica. However, FTIR and nanoindentation results indicated that the H_2_O_2_-cured films possessed a relatively porous network structure, exhibiting lower elastic moduli than furnace-annealed SiO_x_ films and a cage-like Si–O stretching mode. XPS analysis further revealed O/Si ratios of 1.5–1.7 and residual nitrogen contents of 1–2 atomic% [49]. Therefore, the fused-silica-like refractive index of the H_2_O_2_-cured films may reflect the combined effects of their low nitrogen concentration and porous, cage-like Si–O networks.

Intense pulsed UV light (IPL) irradiation produced a glass-like SiO_x_ layer from PHPS in air within 10 min, and the coating surface temperature measured after irradiation remained below 65 °C. For the layer prepared from a 5 wt% PHPS solution, increasing the IPL exposure energy from 4.2 to 12.6 J cm^−2^ reduced the refractive index from 1.49 to 1.45. XPS analysis of the non-etched surface showed that the oxygen content increased, whereas the nitrogen content decreased with increasing exposure energy, indicating progressive oxygen enrichment of the PHPS-derived SiO_x_ layer. Although the refractive index obtained at 12.6 J cm^−2^ was identical to those of amorphous SiO_2_ and the layer heat-treated at 600 °C, residual nitrogen together with Si–N and Si–OH bonding environments remained detectable. Therefore, similarity in refractive index alone does not establish compositional or structural equivalence [60].

**Figure 7:**
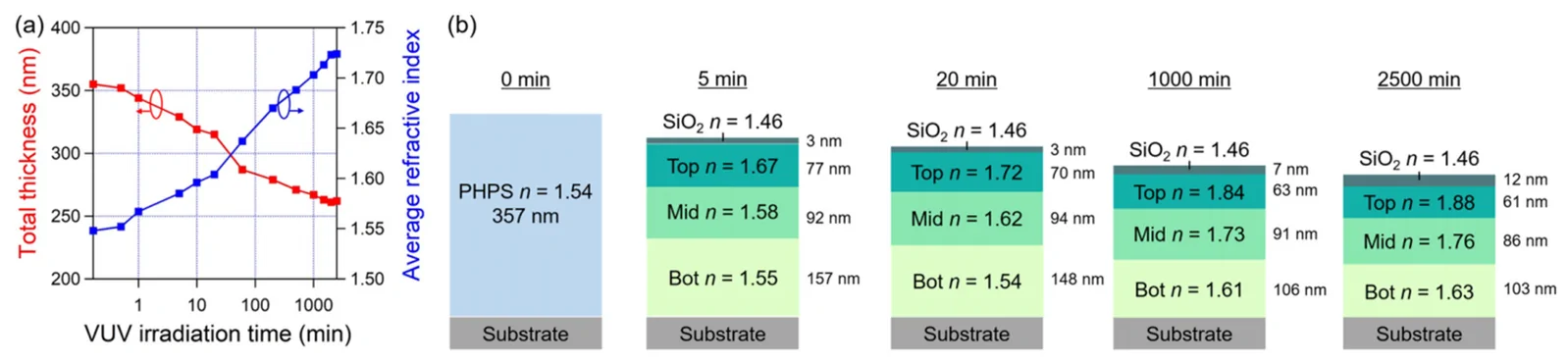
Changes in the thickness and refractive-index characteristics of PHPS films determined by ellipsometry before and after VUV irradiation at 30 °C. (**a**) Total film thickness and mean refractive index as functions of VUV irradiation time. (**b**) Depth-dependent refractive-index distributions of the as-prepared PHPS film and PDSN films after VUV irradiation for 5, 20, 1000, and 2500 min. In panel (**a**), the red and blue arrows indicate that the corresponding curves should be read against the left-hand total-thickness axis and the right-hand average-refractive-index axis, respectively. Reprinted with permission from Ref. [39]. Copyright 2021, The Author(s).

In PDSN films formed under an N_2_ atmosphere, VUV-induced densification was accompanied by an increase in refractive index. With increasing 172 nm VUV irradiation time, the total thickness of the nanometer-thick PDSN films decreased, whereas their average refractive index increased (Figure 7). Analysis using a four-layer ellipsometric model showed that the refractive index of the top layer reached 1.88 after 2500 min of irradiation, remaining below the value of approximately 2.0 for dense stoichiometric Si_3_N_4_ [39]. Notably, although the amount of newly formed Si–N bonds no longer increased beyond approximately 1000 min, the refractive index continued to rise. As discussed in Section 4.2, the increase resulted from repeated Si–N bond cleavage and reformation, leading to network densification and reduced free volume. Even at the same irradiation dose, the refractive index assigned to the top 30 nm region in the four-layer ellipsometric model increased with increasing VUV light intensity, reaching a maximum of 1.90 at 328 mW cm^−2^ and 72 J cm^−2^ [38].

In addition to refractive-index changes, the optical transparency of PHPS-derived films has also been investigated under various conversion conditions. PHPS-derived SiO_x_ layers prepared by IPL irradiation at 12.6 J cm^−2^ preserved the high optical transparency of PET. The layers produced from 3 and 5 wt% PHPS solutions had thicknesses of 93 ± 1.7 and 160 ± 0.7 nm, respectively, with total transmittance values of 90.9% and 90.7%, slightly higher than the 89.0% measured for bare PET [60]. This modest increase was attributed to the reduced refractive index of the coating following PHPS-to-SiO_x_ conversion. Because this value was lower than the refractive index of PET (1.64), the SiO_x_ coating may have improved refractive-index matching between air and the PET substrate, thereby reducing optical reflection and contributing to the slightly higher total transmittance.

The optical transmission of PHPS-derived SiO_x_ layers formed on polymer foils by reel-to-reel (R2R) wet coating followed by VUV conversion was investigated. On a 71 μm-thick PET substrate, SiO_x_ layers with thicknesses of approximately 130–160 nm produced a maximum average relative improvement of 4.2% in direct transmittance over the 400–800 nm wavelength range. This enhancement arose primarily from improved refractive-index matching and reduced reflection through thickness-dependent optical interference. Surface smoothing could additionally reduce light scattering on rough substrates, whereas its contribution was negligible for the initially smooth PET surface [19].

Low-temperature annealing of PHPS at temperatures ranging from 60 to 200 °C for 2 h produced silicon oxynitride coatings on glass with relative transmittance above 98% in the visible region when measured against bare glass. On a 250 μm-thick PET substrate, an approximately 3.18 μm-thick coating formed by annealing at 100 °C for 2 h showed transmittance about 2% higher than that of bare PET throughout the visible region. This increase was explained by the formation of a dense and uniform coating that mitigated surface scratches and defects on the PET, thereby reducing reflection losses [14]. Similarly, VUV irradiation of PHPS-coated PET films (172 nm, 1 h) in dry air formed transparent SiO_x_ coatings below 40 °C while maintaining visible-light transmittance above 80%, which was attributed to reduced surface reflection from the low-refractive-index silica layer [43].

Overall, the optical properties of PHPS-derived films are governed by the degree of conversion together with network composition, bonding structure, and densification. In addition, refractive index and transparency should be interpreted in the context of both the PHPS-derived network and the optical properties of the underlying substrate.

### 5.3. Mechanical and Surface Properties

The mechanical and surface properties of PHPS-derived coatings are influenced by the conversion pathway and extent, residual Si–H and Si–N bonding environments, and the composition and densification of the resulting network. Appropriate conversion processes have been shown to improve hardness and elastic modulus, preserve or further reduce surface roughness, and modify wettability under conditions ranging from room temperature to high-temperature heat treatment [3,11,14,28,49,61].

Among low-temperature conversion methods, aqueous-solution and base-catalyzed treatments have demonstrated the tunability of the mechanical and surface properties of PHPS-derived films [3,11,49]. When spin-coated PHPS films were treated in a 30% aqueous H_2_O_2_ solution at 80 °C for 10 min, their nanoindentation hardness and elastic modulus increased from approximately 0.5 to 3.2 GPa and from 7.0 to 41.6 GPa, respectively, compared with those of the prebaked films. By comparison, the furnace-cured films exhibited a hardness of 13.7 GPa and elastic moduli of 180.6–183.6 GPa. The root-mean-square surface roughness increased slightly from approximately 0.64 to 1.08 nm. Meanwhile, the water contact angle decreased from approximately 93° to 65–80°, indicating increased surface wettability after conversion [49].

PHPS containing 5 wt% APTES was converted at 80 °C, producing a smooth and uniform coating with a volume shrinkage of only 0.86% and an arithmetical mean roughness of 0.293 nm. The coating showed a nanoindentation hardness of 3.62 GPa, an elastic modulus of 30.06 GPa, and a pencil hardness of 9H [3]. Room-temperature exposure to vapor from aqueous ammonia also yielded silica-like films with a pencil hardness exceeding 9H under a 1 kg load [11].

Low-temperature curing has also been investigated as a route for forming PHPS-derived silicon oxynitride coatings. PHPS coatings on glass substrates were annealed at 60–200 °C for 2 h, whereas a PHPS coating on PET film was annealed at 100 °C for 2 h, yielding hard, transparent, and hydrophobic coatings. On glass substrates, the pencil hardness reached 9H, the transmittance remained above 98% across the visible wavelength range when measured relative to bare glass, and the water contact angle ranged from 98° to 110° depending on the annealing temperature. The coating formed on PET at 100 °C showed a pencil hardness of 4H and a water contact angle of 102°, with no obvious delamination after ten repetitions of the cross-cut tape test. No obvious scratches were observed after 50 abrasion cycles using #0000 steel wool under a 2 kg load. These properties indicate that low-temperature annealing produced Si–O–N coatings combining high hardness and scratch resistance, while their hydrophobicity was attributed to residual Si–H bonds in the coating structure [14].

At higher temperatures, thermal conversion was accompanied by more pronounced changes in the mechanical properties and surface morphology of PHPS-derived coatings. PHPS coatings on low-carbon steel were evaluated in the heat-untreated state and after pyrolysis at 200 and 600 °C. The nanoindentation hardness increased from approximately 0.88 GPa in the heat-untreated coating to 1.66 and 5.22 GPa after pyrolysis at 200 and 600 °C, respectively, while Young’s modulus increased from approximately 25.01 GPa to 32.74 and 71.09 GPa. The increases in hardness and Young’s modulus were attributed to progressive network condensation and ceramicization during pyrolysis. In parallel, the average surface roughness decreased from approximately 44.88 nm for the heat-untreated coating to 23.25 nm for the coating pyrolyzed at 600 °C, indicating that increasing pyrolysis temperature was accompanied by mechanical strengthening and surface smoothing [28].

Room-temperature moisture-cured PHPS-based inorganic polysilazane coatings on polycarbonate showed higher hardness and Young’s modulus than polyorganosilazane-derived coatings. However, their water contact angles (84–86°) were lower than those of the polyorganosilazane-derived coatings (95–103°) [61].

Collectively, these studies demonstrate that the mechanical and surface properties of PHPS-derived coatings are governed by the conversion route, processing conditions, and the resulting network structure. Low-temperature catalytic or moisture-assisted conversion can substantially enhance hardness while maintaining smooth surfaces, whereas high-temperature pyrolysis further improves hardness and elastic modulus through continued network condensation. Surface wettability likewise depends on the residual bonding environments, particularly the presence of Si–H groups, indicating that both mechanical performance and surface characteristics are closely linked to the composition and densification of the converted network.

### 5.4. Barrier Properties and Stability

Depending on the conversion conditions, PHPS coatings form SiO-, SiON-, or SiN-based networks that serve as effective barriers against oxygen and water vapor. Their barrier performance and durability are governed by the conversion pathway, the resulting network structure, and processing conditions. This section reviews studies on the gas-barrier performance and durability of PHPS-derived coatings [12,13,17,19,36,38,45,62].

The oxygen-barrier capability of PHPS-derived coatings has been demonstrated on flexible polymer foils. PHPS was applied to a 125 μm PET substrate by R2R slot-die wet coating, followed by low-temperature conditioning at 35 °C for one week to form an approximately 600 nm-thick PHPS-derived SiO_x_ layer. Under measurement conditions of 23 °C, 50% relative humidity, and 100% O_2_, this single-layer coating reduced the OTR from 10.8 cm^3^ m^−2^ day^−1^ bar^−1^ for uncoated PET to 0.09 cm^3^ m^−2^ day^−1^ bar^−1^. Even incompletely converted PHPS-derived SiO_x_ layers substantially reduced oxygen permeation through flexible PET films [19].

However, the barrier performance of PHPS-derived silica formed at low temperatures varied depending on the permeating species. A comparison of low-temperature conversion routes involving damp-heat curing, gaseous or aqueous ammonia, and 20% aqueous H_2_O_2_ showed that ammonia and hydrogen peroxide rapidly promoted cleavage of Si–H and Si–N bonds but did not lead to complete polycondensation of the silanol groups. Consequently, catalyst-assisted conversion produced highly hydroxylated silica, and the resulting layers improved the barrier performance against oxygen and nitrogen, whereas their water-vapor barrier performance remained relatively limited. The lower water-vapor barrier performance was attributed to chemical interactions between residual silanol groups and water and to the high solubility of water in hydroxylated silica [45].

The depth-dependent compositional gradient of UV/VUV-converted PHPS films described in Section 5.1 was reflected in the thickness dependence of their barrier performance. Barrier performance improved as film thickness increased up to approximately 240 nm [17]. The optimum barrier performance was obtained at a thickness corresponding to the converted depth comprising a surface SiO_x_ layer and an underlying SiO_x_Nᵧ layer. The depth of this converted region was limited by oxygen diffusion into the film. The deeper hydrogenated silicon nitride region did not further improve the barrier performance. For films thicker than approximately 240 nm, the barrier performance decreased or the measured values showed greater variability because of cracking induced by mechanical stress. An optimized single layer with a thickness of approximately 240 nm on a 50 μm PET substrate exhibited an OTR of 0.06 cm^3^ m^−2^ day^−1^ bar^−1^ and a WVTR of 0.2 g m^−2^ day^−1^.

In addition to film thickness, the curing method and irradiation distance also had important effects on the barrier performance of PHPS-derived coatings. PHPS was blade-coated onto 125 μm PET substrates, and the resulting films were cured under five different conditions: at room temperature, by dry heating at 100 °C, under damp heat at 65 °C and 85% relative humidity, by 172 nm VUV irradiation, or by simultaneous 172 nm VUV irradiation and heating at 100 °C. FTIR analysis showed that the most rapid PHPS-to-silica conversion occurred when 172 nm VUV irradiation and heating were applied simultaneously at an irradiation distance of 5 mm while maintaining a continuous air flow, with a conversion time of less than 5 min. The WVTR values of the room-temperature-, dry-heat-, and damp-heat-cured films were approximately 4, 2.2, and 1.7 g m^−2^ day^−1^, respectively, whereas that of a single layer cured by simultaneous VUV irradiation and heating at 100 °C decreased to below 0.1 g m^−2^ day^−1^. Moisture-barrier performance was further enhanced by combining simultaneous VUV/thermal curing with multilayer stacking, as demonstrated by a three-layer PHPS-derived structure, in which each layer was cured under the same conditions, exhibiting a WVTR below 0.002 g m^−2^ day^−1^ [15]. Similar photo-thermal conversion was also reported for alicyclic polyimide (PI) and heat-resistant PET substrates [63,64]. In particular, a PHPS-derived barrier film formed on heat-resistant PET by combining 172 nm VUV irradiation from an excimer lamp with substrate heating at 150 °C reduced the WVTR to below 0.02 g m^−2^ day^−1^ and also exhibited high transparency, good adhesion, low surface roughness, and flexibility [64].

PDSN layers formed by VUV irradiation under an N_2_ atmosphere achieved lower WVTR values than the SiO_x_-based barriers discussed above, although direct comparison is limited because the measurement conditions differed among studies. Three alternating polydimethylsiloxane (PDMS)/PDSN bilayers were deposited on a PI substrate to form a PI/(PDMS/PDSN)_3_ structure, in which the PDSN layers were approximately 200 nm thick and the PDMS layers served as stress-relaxation layers. This structure exhibited a WVTR of 4.8 × 10^−5^ g m^−2^ day^−1^ [13]. Subsequently, the same PI/(PDMS/PDSN)_3_ structure was processed using high-power VUV irradiation at 309 mW cm^−2^. At a dose of 6 J cm^−2^, an average WVTR of 1.6 × 10^−5^ g m^−2^ day^−1^ was achieved with an irradiation time of 20 s per PHPS layer. Beyond this optimal dose, the barrier performance gradually deteriorated [38]. The fabrication environment before VUV irradiation also affected the barrier performance of PDSN layers. Under dry conditions, increasing the oxygen concentration from 0% to 20% produced almost no change in the chemical structure of the PHPS film over 24 h. By contrast, ambient humidity induced hydrolysis before irradiation, resulting in decreased Si–N bonding and increased Si–O incorporation after the subsequent VUV treatment. When the PHPS layers were exposed to each humidity condition for 5 min before VUV irradiation, the WVTR of the PI/(PDMS/PDSN)_3_ structure increased from 1.7 × 10^−5^ g m^−2^ day^−1^ at 0% relative humidity to 4.7 × 10^−5^ and 5.4 × 10^−4^ g m^−2^ day^−1^ at 20% and 60% relative humidity, respectively. These results show that humidity control is more critical than oxygen-concentration control for achieving reproducible ultrahigh barrier performance [36].

PHPS-derived silica showed high hot-water durability even after room-temperature conversion. After PHPS films were exposed to vapor from aqueous ammonia for 6 h and then soaked in water at 80 °C for 24 h, their thickness decreased by about 2%. This reduction was substantially smaller than the thickness losses of 65% and 98% observed for ambient-dried TEOS-derived silica gel films and those exposed to ammonia vapor, respectively. It was also comparable to the 4% reduction measured for a TEOS-derived film fired at 500 °C. These results indicate that room-temperature conversion of PHPS yielded hydrolytically stable silica films without high-temperature firing [62].

Overall, the barrier performance improved as the converted network became denser and more continuous, whereas residual hydroxyl groups, compositional gradients, and stress-induced cracking limited moisture-barrier performance.

## 6. Applications of PHPS-Derived Materials

### 6.1. Applications of SiN-Based Materials

Silicon nitride (SiN_x_) materials derived from PHPS exhibit excellent moisture-barrier performance, thermal stability, and mechanical strength, making them attractive for a wide range of industrial applications.

#### 6.1.1. Barrier Layers for Flexible Electronics and Packaging

One of the most extensively investigated applications of PDSN is thin-film encapsulation technology for the protection of next-generation flexible electronic devices, including OLEDs, PSCs, and organic photovoltaic (OPV) devices [36,37,39]. Organic and perovskite-based devices are highly susceptible to degradation caused by moisture and oxygen, which can induce electrode corrosion and the formation of dark spots, ultimately deteriorating device performance. To ensure long-term operational stability, barrier layers with extremely low WVTRs are required. In general, WVTR values below 10^−5^ g m^−2^ day^−1^ for OLEDs and below 10^−3^ g m^−2^ day^−1^ for PSCs are considered necessary for effective device protection [13,38].

Conventional vacuum-based deposition processes, such as ALD and plasma-enhanced chemical vapor deposition, provide excellent barrier performance but are associated with high equipment costs, complex processing cycles, and limited compatibility with R2R manufacturing [36]. In contrast, PHPS-based solution processing enables low-cost, large-area fabrication under atmospheric conditions while offering high throughput.

SiN_x_ thin films cured at room temperature through vacuum ultraviolet (VUV) irradiation have demonstrated barrier performance comparable to that of glass [13]. In particular, organic/inorganic multilayer architectures consisting of alternating SiN_x_ barrier layers and compliant organic interlayers (e.g., PDMS) have been widely adopted to simultaneously achieve excellent barrier performance and mechanical flexibility [36,38]. The barrier performance of PHPS-derived SiN_x_ films is closely related to film density and refractive index (n), which are commonly used as key indicators of film quality. Accordingly, considerable efforts have been devoted to optimizing curing conditions and processing parameters to maximize moisture-barrier performance [37,39].

Beyond thin-film encapsulation for flexible electronics, PHPS-derived SiN_x_ barrier films are also promising for packaging applications requiring effective protection against oxygen and moisture. Their excellent gas barrier performance makes them attractive as coating materials for packaging systems requiring effective suppression of food oxidation and maintenance of pharmaceutical stability, although packaging-specific oxygen transmission, sealing performance, product compatibility, and shelf-life extension were not evaluated [13,39]. Furthermore, the compatibility of PHPS with solution-based processing and printing technologies enables the direct patterning of gas barrier layers without relying on complex vacuum-based fabrication processes. This capability offers significant advantages for printed electronics and advanced packaging applications, where low-cost, large-area, and high-throughput production is increasingly required.

#### 6.1.2. High-Strength Ceramic Fibers

PHPS has been widely utilized as a precursor for the fabrication of silicon nitride ceramic fibers, which exhibit excellent thermal stability and mechanical strength [40,41]. Silicon nitride fibers exhibit high hardness, outstanding thermal shock resistance, excellent strength retention at elevated temperatures, and superior corrosion resistance against molten non-ferrous metals such as aluminum. In addition, they possess excellent electrical insulation properties and a Young’s modulus higher than that of carbon steel. The mechanical performance of PDSN fibers is strongly influenced by the pyrolysis temperature. High-performance fibers with tensile strengths of up to approximately 4300 MPa have been obtained at pyrolysis temperatures around 1400 °C [40]. Their exceptional mechanical and thermal properties make these fibers well suited as reinforcing materials for composite systems based on plastics, metals, glass, and ceramics. Consequently, they have attracted considerable interest for structural applications in aerospace systems and other high-temperature industrial environments [65].

### 6.2. Applications of SiO-Based Materials

#### 6.2.1. Gas Barrier Layers for Flexible Electronics

One of the most important applications of PHPS-derived silica is the fabrication of high-performance gas barrier layers on thermally sensitive polymer substrates, such as PET, PEN, and biodegradable cellulose-based films [8,59,64,66,67,68,69,70]. As discussed in Section 3.1.1, OLEDs, OPVs, and PSCs are highly susceptible to degradation caused by moisture and oxygen exposure [71,72,73,74,75,76,77,78,79]. Coating of PHPS followed by conversion through VUV irradiation or low-temperature thermal curing yields silica barrier films exhibiting excellent moisture barrier performance, with WVTR values below 10^−3^ g m^−2^ day^−1^ [15].

To demonstrate the practical applicability of PHPS-derived encapsulation layers, flexible PSCs fabricated on PEN substrates were encapsulated with a quantum dot/PHPS barrier layer. The encapsulated devices retained 99.2% of their initial efficiency after 400 h under ambient conditions, whereas unencapsulated devices retained only 77.9%, demonstrating the effectiveness of PHPS-based encapsulation in enhancing the environmental stability of flexible PSCs [80]. The solution-processability of PHPS makes it particularly attractive for R2R coating processes, enabling low-cost, large-area fabrication with high throughput and thereby enhancing its potential for industrial-scale production [19].

To further improve barrier performance, multilayer architectures consisting of alternating inorganic layers (PHPS-derived silica) and organic interlayers have been investigated. In these structures, the organic layers compensate for microscopic defects and pinholes present in the inorganic barrier layers, creating a tortuous diffusion pathway [81,82] for permeating gas molecules and thereby significantly suppressing gas transport through the coating [15,19,83,84].

#### 6.2.2. Semiconductor Insulators and Electronic Components

**Figure 8:**
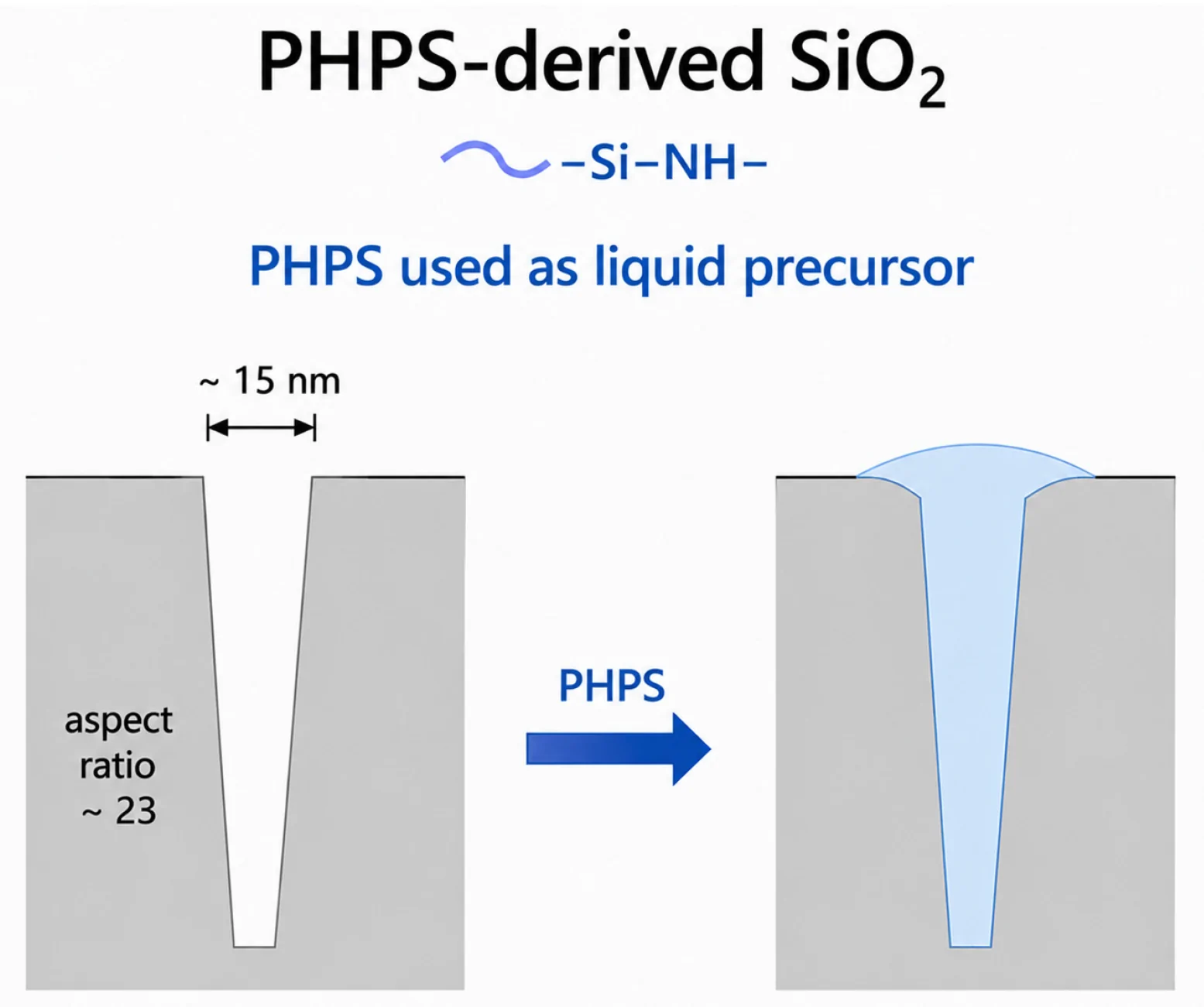
Schematic illustration of the gap-filling capability of PHPS as a liquid precursor for semiconductor STI applications. PHPS enables void-free filling of extremely narrow and high-aspect-ratio trenches (~23). Gray represents the patterned substrate, white represents the empty trench before filling, and light blue represents the PHPS-derived SiO_2_ filling the trench.

In semiconductor manufacturing, PHPS-derived silica has been utilized as an interlayer dielectric and STI material [58,85,86]. As device dimensions continue to shrink, conventional CVD-based silica deposition has encountered increasing difficulties in completely filling narrow and high-aspect-ratio structures, often resulting in the formation of voids and seams [87,88,89,90]. In contrast, PHPS can be applied as a liquid precursor with excellent flowability, enabling void-free filling of extremely narrow, high-aspect-ratio patterns with widths of ~15 nm and aspect ratios of ~23 (Figure 8) [21]. This superior gap-filling capability has made PHPS-derived silica an attractive material for advanced semiconductor fabrication processes.

In addition to void-free filling, excellent electrical insulation properties are required for dielectric applications. PHPS-derived silica subjected to high-temperature thermal treatment exhibits properties comparable to those of bulk silica glass, including a density of 2.1–2.2 g cm^−3^, a refractive index of 1.45–1.46, and an electrical resistivity of approximately 10^15^ Ω·cm [11,12,16]. These characteristics make PHPS-derived silica highly suitable for semiconductor insulating applications. Furthermore, low-temperature VUV-assisted conversion has enabled the use of PHPS-derived silica as a gate dielectric for metal oxide thin-film transistors. Devices incorporating PHPS-derived dielectric layers have demonstrated high charge-carrier mobility together with excellent operational reliability, highlighting the potential of PHPS-based materials for electronic devices [58].

#### 6.2.3. Coatings

##### Optical and Functional Coatings

PHPS-derived silica has attracted considerable attention for optical coating applications because of its combination of high transparency and tunable refractive index [91]. In particular, PHPS-derived silica has been employed as the dielectric layer in multilayer metal–dielectric structures to generate vivid and durable structural colors. Unlike conventional pigment- or dye-based coloration, this approach offers an environmentally friendly alternative with excellent long-term color stability. Consequently, such coatings have been considered promising for decorative applications in automotive exterior components and premium consumer electronics [48].

In addition, PHPS-derived silica has been utilized as an antireflective coating by precisely controlling the film thickness and refractive index, thereby reducing substrate reflectance and enhancing light transmittance [15]. Burak and co-workers further demonstrated that the formation of an ultrathin silica shell on TiO_2_ particles effectively suppresses the intrinsic photocatalytic activity of TiO_2_ while preserving its high whiteness and refractive index. This strategy minimizes discoloration and potential skin irritation, making the coated particles attractive for cosmetic and paint pigment applications [91]. Yamano and Kozuka also reported the successful incorporation of high concentrations of spiropyran, a photochromic molecule capable of reversible structural transformation upon light irradiation, into silica films [92]. The resulting coatings exhibited reversible color changes induced by photochromic reactions, demonstrating the potential of PHPS-derived silica as a platform for functional optical coatings and stimuli-responsive materials.

##### Protective and Anticorrosion Coatings

PHPS-derived silica coatings have been widely investigated as protective layers for improving surface hardness and preventing oxidation and corrosion of metallic and polymeric substrates. One notable application is the protection of aerospace materials from atomic oxygen attack in low-Earth orbit environments. PHPS-derived coatings have been applied to polymeric materials such as Kapton, where they provide excellent erosion resistance against atomic oxygen exposure [26]. These coatings exhibit minimal volumetric shrinkage during oxidation, thereby reducing crack formation. In addition, the presence of residual free silicon enables a self-healing mechanism through oxidation, which further enhances their long-term durability under harsh environmental conditions. In conventional industrial applications, PHPS-derived coatings have been deposited on metallic substrates, including stainless steel (SUS) and aluminum alloys, to improve resistance against high-temperature oxidation and chemical corrosion [93,94,95]. Protective coatings based on PHPS have also been developed for Mo–Si–B alloys, which inherently exhibit limited oxidation resistance. By incorporating Si–B fillers into the PHPS matrix, dense protective layers capable of withstanding temperatures above 1100 °C have been produced [65,96,97,98].

Interestingly, PHPS-derived coatings have also been explored as transparent protective layers for the conservation of historical glass artifacts by suppressing corrosion caused by atmospheric moisture and environmental contaminants [99]. Furthermore, their excellent chemical stability and corrosion resistance have motivated investigations into their application as protective coatings for steel canisters intended for the long-term storage of radioactive nuclear waste in highly corrosive environments [24].

#### 6.2.4. Dental and Healthcare Materials

**Figure 9:**
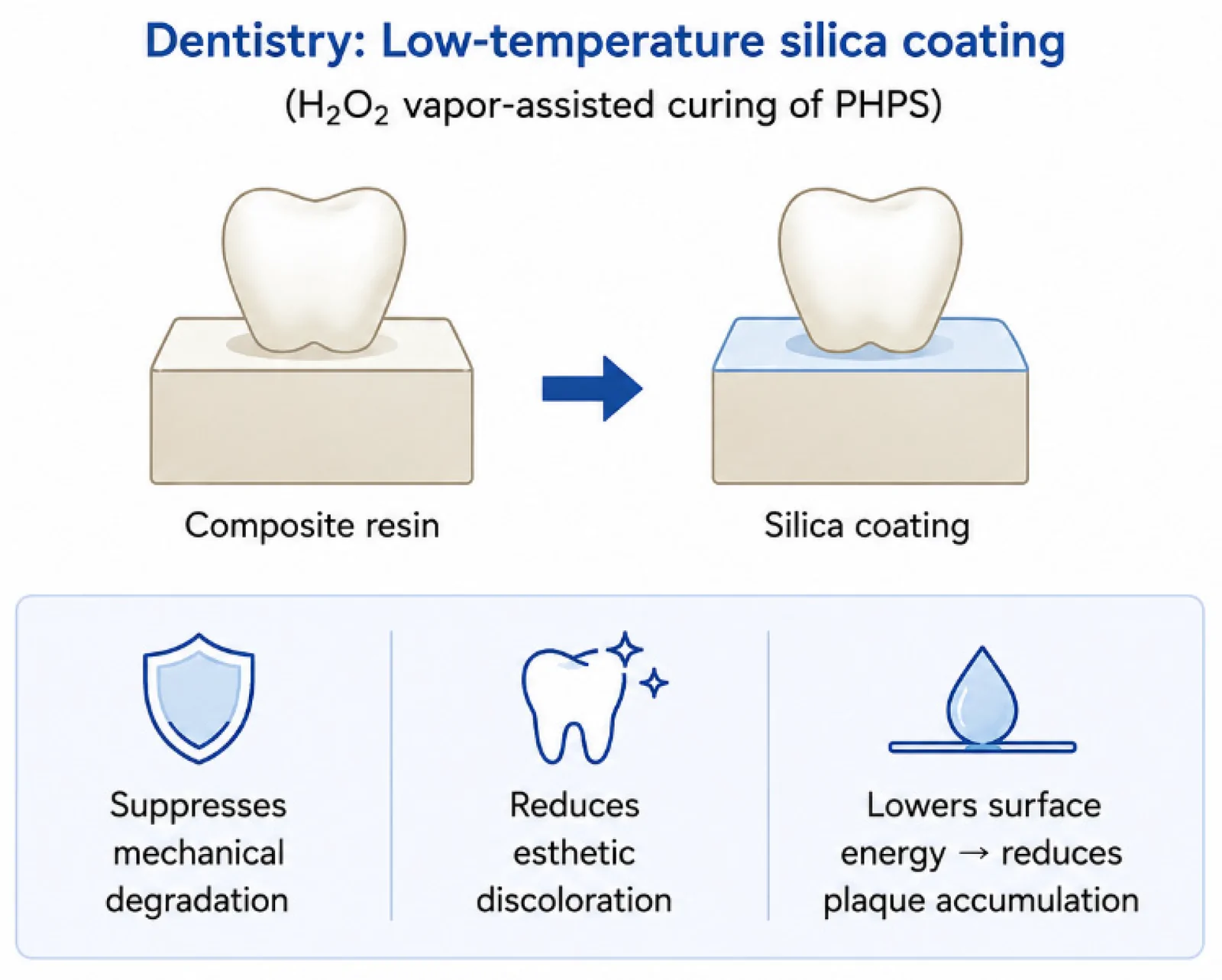
Schematic illustration of low-temperature silica coating on dental composite resin using PHPS. The PHPS-derived silica coating improves the durability and esthetic stability of the resin while reducing plaque accumulation through decreased surface energy. The arrow indicates the application of a PHPS-derived silica coating to the initially uncoated composite-resin surface through H_2_O_2_ vapor-assisted curing.

PHPS-derived silica has attracted considerable attention for healthcare applications because of its excellent biocompatibility, chemical stability, and resistance to contamination. In dentistry, low-temperature silica coatings have been formed on composite resin surfaces through hydrogen peroxide vapor-assisted curing of PHPS. These coatings effectively suppress the mechanical degradation of the resin, significantly reduce esthetic discoloration during long-term use, and lower the surface energy of the resin, thereby reducing plaque accumulation (Figure 9) [44]. In addition, Ag-containing PHPS-derived silica coatings inhibit *Escherichia coli* growth and exhibit strong antibacterial activity, further expanding their potential for biomedical applications [48].

#### 6.2.5. Surface Modification and Other Applications

##### Hydrophilic and Superhydrophobic Surfaces

PHPS-derived silica coatings have been employed for surface wettability control in a variety of applications. The intrinsic hydrophilicity of PHPS-derived silica coatings has been utilized to enhance water vapor transport during dehumidification processes [100]. In contrast, when PHPS-derived silica is deposited onto laser-fabricated micro/nanostructured surfaces, the resulting coatings exhibit extremely low surface energy and hierarchical surface roughness, leading to superhydrophobic behavior in which water droplets readily roll off the surface without wetting it [101].

##### Gas Separation Membranes

Pyrolysis of PHPS can produce inorganic membranes with pore sizes in the sub-nanometer range. These membranes exhibit high hydrogen-selective permeability and have therefore attracted considerable interest for hydrogen separation processes and membrane reactor applications [102,103,104,105].

##### Luminescent Films

Functional luminescent films have been fabricated by homogeneously dispersing nanophosphors such as ZnS:Mn^2+^ within a PHPS matrix. The resulting composite films maintain both high optical transparency and efficient photoluminescence, demonstrating their potential for optical and display-related applications [46].

##### Wafer Bonding

PHPS has also been investigated as an interfacial material for silicon wafer bonding. Plasma treatment converts the PHPS surface into a highly hydrophilic state, facilitating strong wafer-to-wafer bonding even at room temperature [106]. This approach offers a low-temperature alternative to conventional wafer-bonding processes and is particularly attractive for applications involving thermally sensitive devices and substrates.

### 6.3. Applications of SiON-Based Materials

Although the application range of SiON-based materials is less extensive than that of SiO-based materials, they have attracted considerable interest because of their high transparency, hardness, chemical stability, and moisture-barrier performance. As a result, PHPS-derived SiO_x_N_y_ materials have been investigated for a variety of protective and functional applications.

#### 6.3.1. Protective Coatings and Barrier Layers

##### Stone Conservation

Sandstone is particularly vulnerable to water ingress and subsequent salt crystallization because of its porous microstructure. PHPS-derived SiO_x_N_y_ coatings form dense and uniform protective layers through chemical bonding with hydroxyl groups present on the sandstone surface. Compared with conventional protective treatments based on acrylic resin B72 or siloxane materials such as PDMS [107,108], SiO_x_N_y_ coatings significantly reduce water absorption and porosity while retaining nearly 85% of the original water-vapor permeability of the stone [50]. Furthermore, the coatings mitigate salt-induced deterioration and cause minimal changes in the appearance of colored sandstone, making them attractive for stone conservation applications.

##### Semiconductor Packaging

PHPS-derived SiO_x_N_y_ layers have been employed as interfacial coatings between Cu and EMCs to improve package reliability. In semiconductor packaging, Cu/EMC interfaces are susceptible to moisture ingress, chloride-induced corrosion, and interfacial delamination [109,110]. By spin-coating PHPS onto Cu surfaces followed by thermal curing, ultrathin SiO_x_N_y_ layers with thicknesses of only several tens of nanometers can be formed. These layers enhance interfacial adhesion through covalent bonding with Cu and hydrogen bonding as well as mechanical interlocking with EMC, resulting in adhesion-strength improvements of up to 57%. Furthermore, the SiO_x_N_y_ layer acts as an effective barrier against moisture and ionic species, maintaining stable interfacial bonding even under salt-water immersion conditions while having a negligible impact on heat dissipation because of its extremely small thickness [51].

##### Hard Coatings for Optical Devices

PHPS-derived SiO_x_N_y_ coatings have been investigated as protective coatings for optical components, such as lenses, displays, and touch panels, because of their high transparency and hardness. Thermal curing of PHPS at temperatures below 200 °C produces transparent SiO_x_N_y_ coatings with visible-light transmittance of 90–98%, thereby avoiding high-temperature processes that may damage temperature-sensitive substrates. The resulting coatings exhibit excellent hardness and scratch resistance. In addition, residual Si–H bonds remaining after low-temperature curing impart hydrophobicity to the coating surface, providing antifouling properties and facilitating surface cleaning [14].

##### Solar Cell Encapsulation

PHPS-derived SiO_x_N_y_ coatings have been investigated as encapsulation materials for flexible solar cells and organic electronic devices to improve their operational stability [54,55]. Because perovskite and organic solar cells are highly susceptible to degradation induced by moisture and oxygen, laminated composite coatings incorporating single-walled carbon nanotubes (SWCNTs) within a SiO_x_N_y_ matrix have been developed. In addition to the excellent barrier performance of the SiO_x_N_y_ layer, the embedded SWCNTs act as reinforcing components that suppress coating failure and enhance mechanical durability. Furthermore, the compositional gradient formed during the curing process provides an antireflection effect over a broad wavelength range, thereby increasing light transmission into the solar cell.

#### 6.3.2. Wave-Transparent Composites

In high-temperature aerospace composites, excessively strong interfacial bonding between reinforcing fibers and the matrix can lead to brittle failure. In addition, thermal stresses may develop because of the mismatch in coefficients of thermal expansion between the constituent materials. To address these issues, SiO_x_N_y_ has been employed as an interfacial modification layer between silicon nitride (Si_3_N_4_) fibers and silica (SiO_2_) matrices [52,53]. The SiO_x_N_y_ coating provides a healing effect by filling surface flaws on the fibers, resulting in an approximately 24% increase in tensile strength. Its intermediate coefficient of thermal expansion helps alleviate thermally induced stresses at the fiber–matrix interface. At the same time, SiO_x_N_y_ maintains favorable dielectric properties, allowing the composite to retain excellent electromagnetic-wave transparency.

## 7. Conclusions and Outlook

PHPS should be regarded not merely as a precursor to silica but as a tunable preceramic platform for producing compositionally and structurally diverse silicon-based networks. Depending on the conversion route, PHPS-derived materials span a broad range of structures, including SiO_x_-rich and silica-like networks, SiO_x_Nᵧ intermediates, depth-graded oxide/oxynitride/nitride architectures, N_2_/VUV-derived PDSN films, amorphous Si_3_N_4_-like networks, and crystalline Si_3_N_4_-containing ceramics. This versatility arises from the solution processability of the polymeric precursor and the sensitivity of its conversion behavior to atmospheric composition, temperature, humidity, the presence of catalysts or reactive media, irradiation parameters, film thickness, and post-treatment history. The principal value of PHPS therefore lies not in its conversion into a single final composition but in the possibility of directing the precursor toward different inorganic network states through controlled chemical, thermal, and photochemical processing.

PHPS conversion cannot be described by a single oxidation or ceramization process. Instead, hydrolysis, oxidation, dehydrogenation, nitridation, photon-induced bond cleavage, and reactive-species-mediated processes contribute to different extents depending on the processing environment. H_2_O primarily promotes hydrolysis and nitrogen removal, whereas O_2_ facilitates Si–O bond formation but may become diffusion-limited after the formation of an oxide-rich surface layer. Under NH_3_ or N_2_ atmospheres, nitridation and Si–N network reconstruction are favored, while VUV-assisted conversion is governed by the balance between direct photolysis and reactive-species-mediated oxidation. Furthermore, limited photon penetration and reactive-species transport can produce depth-dependent conversion, particularly in thicker films. Consequently, the final network structure is determined by the coupled effects of reaction pathways, species transport, and network reconstruction rather than by the nominal conversion method alone.

The properties of PHPS-derived coatings are governed by the resulting network structure rather than by their nominal classification as SiO_x_, SiO_x_Nᵧ, or SiN_x_. Residual bonding environments, network connectivity, compositional gradients, defects, and coating–substrate interactions collectively determine their optical, mechanical, surface, and barrier properties. Consequently, optimizing PHPS-derived coatings requires balanced control of network structure, surface chemistry, effective thickness, interfacial bonding, and defect formation rather than maximizing any single processing parameter or material property.

This structural tunability has supported the investigation of PHPS-derived materials across several application classes. PDSN-based thin films and organic/inorganic multilayers have shown moisture-barrier performance relevant to flexible-electronics encapsulation, while SiO_x_-rich coatings have been evaluated as transparent oxygen- and water-vapor barriers on polymer substrates. Oxygen-containing networks have also been investigated as dielectric and interfacial layers, transparent hard coatings, protective and anticorrosion coatings, stone-conservation treatments, surface modifiers with controlled wettability, and functional composite interphases. High-temperature conversion further extends the material platform to Si_3_N_4_-based fibers and ceramic structures.

Nevertheless, the level of validation differs substantially among these fields. Several studies directly measured film properties, permeation rates, interfacial adhesion, or device performance, whereas other applications were proposed primarily on the basis of favorable material properties. Standalone coating performance should therefore not automatically be regarded as evidence of long-term device reliability or industrial implementation. Differences in precursor formulation, film thickness, substrate, conversion conditions, and test protocols also limit direct comparison among reported values. Many proposed applications still lack repeated bending tests, thermal and humidity cycling, interfacial-aging studies, component-level validation, and long-term operational testing.

Future progress requires a transition from empirical optimization toward predictive control of PHPS conversion. Processing maps should correlate precursor formulation and processing parameters with network composition, densification, and depth-dependent structure. Achieving this goal will require the integration of in situ spectroscopy, depth-resolved chemical analysis, quantitative characterization of density and free volume, and multiscale modeling to elucidate the relationships among bond cleavage, species transport, and network reconstruction. Equally important is the establishment of standardized processing and characterization protocols to enable meaningful comparison and reproducible development of PHPS-derived materials.

Building on these advances, future research should extend toward sustainable and scalable manufacturing strategies. Owing to its compatibility with solution processing and low-temperature conversion, PHPS offers considerable potential to reduce the energy consumption associated with conventional vacuum-based ceramic coating technologies. These advantages should be quantitatively assessed through life-cycle assessment (LCA) and carbon-footprint analysis, while environmentally benign, metal-catalyst-free processing routes should be developed to minimize contamination. Finally, future studies should move beyond film-level characterization toward comprehensive device-level validation, including long-term reliability, environmental stability, and large-area manufacturing. Advancing predictive conversion, sustainable processing, standardized evaluation, and device validation will be essential for translating the structural tunability of PHPS into practical silicon-based technologies.

## Data Availability

No new data were created or analyzed in this study. Data sharing is not applicable to this article.

## Abbreviations

The following abbreviations are used in this manuscript:

ALD	atomic layer deposition
APTES	3-aminopropyltriethoxysilane
CVD	chemical vapor deposition
DCS	dichlorosilane
DMAPO	2-dimethylamino-1-propanol
DUV	deep-ultraviolet
EMC	epoxy molding compound
FTIR	Fourier-transform infrared spectroscopy
IPL	intense pulsed UV light
NMR	nuclear magnetic resonance
OLEDs	organic light-emitting diodes
OPV	organic photovoltaic
OTR	oxygen transmission rate
PDSN	PHPS-derived silicon nitride
PDMS	polydimethylsiloxane
PEN	poly(ethylene naphthalate)
PET	poly(ethylene terephthalate)
PHPS	perhydropolysilazane
PI	polyimide
PSC	perovskite solar cell
R2R	reel-to-reel
ReaxFF	reactive force field
ROS	reactive oxygen species
STI	shallow trench isolation
SWCNT	single-walled carbon nanotube
TEOS	tetraethyl orthosilicate
TOF-SIMS	time-of-flight secondary ion mass spectrometry
UV	ultraviolet
VTES	vinyltriethoxysilane
VUV	vacuum ultraviolet
WVTR	water vapor transmission rate
XPS	X-ray photoelectron spectroscopy

## References

[1] Schwab S.T., Graef R.C., Blanchard C.R., Dec S.F., Maciel G.G. (1998). The pyrolytic conversion of perhydropolysilazane into silicon nitride. Ceram. Int..

[2] Hou D., Chen J., Li Y., Li P., Zhang Z., Jiang J. (2025). Atmosphere-regulated conversion of perhydropolysilazane (PHPS): Controlling Si–O–N composition and unveiling atomistic mechanisms. ACS Appl. Mater. Interfaces.

[3] Wang W.-Y., Zhang Y.-L., Guo X., Wang L.-M., Zhang J.-R., Yang H., Dong G.-J., Zhang Z.-B., Xu C.-H. (2023). Rapid conversion of perhydropolysilazane into thin silica coating at low temperature. Chin. J. Polym. Sci..

[4] Barroso G., Li Q., Bordia R.K., Motz G. (2019). Polymeric and ceramic silicon-based coatings—A review. J. Mater. Chem. A.

[5] Chatterjee A., Sen S., Ramakanth D., Singh S., Maji P.K. (2025). Unravelling polysilazanes: Synthesis, structure-property insights and versatile coating applications. Adv. Colloid Interface Sci..

[6] Naganuma Y., Kato C., Watanabe T., Kaneko S., Tanaka S. (2024). Physicochemical and structural properties of silica films prepared from perhydropolysilazane using vacuum ultraviolet irradiation. Thin Solid Films.

[7] Funayama O., Tashiro Y., Kamo A., Okumura M., Isoda T. (1994). Conversion mechanism of perhydropolysilazane into silicon nitride-based ceramics. J. Mater. Sci..

[8] Prager L., Dierdorf A., Liebe H., Naumov S., Stojanović S., Heller R., Wennrich L., Buchmeiser M.R. (2007). Conversion of perhydropolysilazane into a SiO_x_ network triggered by vacuum ultraviolet irradiation: Access to flexible, transparent barrier coatings. Chem. Eur. J..

[9] Colombo P., Mera G., Riedel R., Sorarù G.D. (2010). Polymer-derived ceramics: 40 years of research and innovation in advanced ceramics. J. Am. Ceram. Soc..

[10] Riedel R., Mera G., Hauser R., Klonczynski A. (2006). Silicon-based polymer-derived ceramics: Synthesis, properties and applications—A review. J. Ceram. Soc. Jpn..

[11] Nakajima K., Uchiyama H., Kitano T., Kozuka H. (2013). Conversion of solution-derived perhydropolysilazane thin films into silica in basic humid atmosphere at room temperature. J. Am. Ceram. Soc..

[12] Kubo T., Kozuka H. (2006). Conversion of perhydropolysilazane-to-silica thin films by exposure to vapor from aqueous ammonia at room temperature. J. Ceram. Soc. Jpn..

[13] Sasaki T., Sun L., Kurosawa Y., Takahashi T., Suzuri Y. (2022). Solution-processed gas barriers with glass-like ultrahigh barrier performance. Adv. Mater. Interfaces.

[14] Zhang Z., Shao Z., Luo Y., An P., Zhang M., Xu C. (2015). Hydrophobic, transparent and hard silicon oxynitride coating from perhydropolysilazane. Polym. Int..

[15] Channa I.A., Shah A.A., Rizwan M., Makhdoom M.A., Chandio A.D., Shar M.A., Mahmood A. (2021). Process parameter optimization of a polymer derived ceramic coatings for producing ultra-high gas barrier. Materials.

[16] Kozuka H., Nakajima K., Uchiyama H. (2013). Superior properties of silica thin films prepared from perhydropolysilazane solutions at room temperature in comparison with conventional alkoxide-derived silica gel films. ACS Appl. Mater. Interfaces.

[17] Morlier A., Cros S., Garandet J.P., Alberola N. (2014). Structural properties of ultraviolet cured polysilazane gas barrier layers on polymer substrates. Thin Solid Films.

[18] Kubo T., Tadaoka E., Kozuka H. (2004). Formation of silica coating films from spin-on polysilazane at room temperature and their stability in hot water. J. Mater. Res..

[19] Blankenburg L., Schrödner M. (2015). Perhydropolysilazane derived silica for flexible transparent barrier foils using a reel-to-reel wet coating technique: Single- and multilayer structures. Surf. Coat. Technol..

[20] Naganuma Y., Tanaka S., Kato C., Shindo T. (2004). Formation of silica coatings from perhydropolysilazane using vacuum ultraviolet excimer lamp. J. Ceram. Soc. Jpn..

[21] Kim S.-D., Ko P.-S., Park K.-S. (2013). Perhydropolysilazane spin-on dielectrics for inter-layer-dielectric applications of sub-30 nm silicon technology. Semicond. Sci. Technol..

[22] Park K.-S., Ko P.-S., Kim S.-D. (2014). Effects of N_2_O plasma treatment on perhydropolysilazane spin-on-dielectrics for inter-layer-dielectric applications. Thin Solid Films.

[23] Kim G.-H., Park S.-Y., Pyo S.-S., Han J.-H., Kim J.-N., Oh K.-J., Ryu C.-K., Choi Y.-S., Kwak N.-J., Park S.-K. (2009). Effect of wet treatment on stability of spin-on dielectrics for STI gap-filling in nanoscale memory. Solid State Phenom..

[24] Wang W., Tamakloe S., Deng Z., Li L., Cai W., Lu K. (2022). Effects of processing temperature on the corrosion and tribocorrosion resistance of perhydropolysilazane-derived coatings on AISI 304 steel. Surf. Coat. Technol..

[25] Choi H.J., Lu K. (2024). Polysilazane-derived SiON coating on stainless steel weld for corrosion resistance. Mater. Chem. Phys..

[26] Hu L., Li M., Xu C., Luo Y. (2011). Perhydropolysilazane derived silica coating protecting Kapton from atomic oxygen attack. Thin Solid Films.

[27] Yang N., Xu S., Xu C. (2022). Highly electromagnetic transparent ceramic composite made of boron nitride nanotubes and silicon oxynitride *via* perhydropolysilazane infiltration method. Sci. Rep..

[28] Yang N., Wang W., Cai W., Lu K. (2020). Corrosion and tribocorrosion mitigation of perhydropolysilazane-derived coatings on low carbon steel. Corros. Sci..

[29] Stock A., Somieski K. (1921). Silicon Hydrides, X: Nitrogen-containing compounds. Berichte Dtsch. Chem. Ges..

[30] Seyferth D., Wiseman G.H., Prud’homme C. (1983). A liquid silazane precursor to silicon nitride. J. Am. Ceram. Soc..

[31] Campbell-Ferguson H.J., Ebsworth E.A.V. (1966). Adducts formed between some halogenosilanes and the organic bases pyridine, trimethylamine, and tetramethylethylenediamine. Part I. Stoichiometry. J. Chem. Soc. A Inorg. Phys. Theor..

[32] Hensen K., Stumpf T., Bolte M., Näther C., Fleischer H. (1998). Experimental Investigations and ab Initio Studies on hexacoordinated complexes of dichlorosilane. J. Am. Chem. Soc..

[33] Scantlin W.M., Norman A.D. (1972). Borane-catalyzed condensation of trisilazane and N-methyldisilazane. Inorg. Chem..

[34] Isoda T., Arai M. (1985). Synthesis of Inorganic Polysilazane. JP Patent.

[35] Isoda T., Kaya H., Nishii H., Funayama O., Suzuki T., Tashiro Y. (1992). Perhydropolysilazane precursors to silicon nitride ceramics. J. Inorg. Organomet. Polym..

[36] Song L., Sun H., Suzuri Y. (2025). Degradation driven by ambient oxygen and moisture during film preparation of solution-processed PHPS barrier films. Appl. Phys. Express.

[37] Song L., Sun H., Suzuri Y. (2025). Morphological observation of photo-densified PHPS under 172 nm VUV irradiation at varying light intensities for moisture barrier applications. Jpn. J. Appl. Phys..

[38] Song L., Sun H., Suzuri Y. (2025). Polysilazane-coated films achieving record-high moisture barrier performance with sub-10 seconds densification using high-power VUV irradiation. Adv. Sci..

[39] Sasaki T., Sun L., Kurosawa Y., Takahashi T., Suzuri Y. (2021). Nanometer-thick SiN films as gas barrier coatings densified by vacuum UV irradiation. ACS Appl. Nano Mater..

[40] Funayama O., Arai M., Tashiro Y., Aoki H., Suzuki T., Tamura K., Kaya H., Nishii H., Isoda T. (1990). Tensile strength of silicon nitride fibers produced from perhydropolysilazane. J. Ceram. Soc. Jpn..

[41] Yokoyama Y., Nanba T., Yasui I., Kaya H., Maeshima T., Isoda T. (1991). X-ray diffraction study of the structure of silicon nitride fiber made from perhydropolysilazane. J. Am. Ceram. Soc..

[42] Asakuma N., Tada S., Kawaguchi E., Terashima M., Honda S., Nishihora R.K., Carles P., Bernard S., Iwamoto Y. (2022). Mechanistic investigation of the formation of nickel nanocrystallites embedded in amorphous silicon nitride nanocomposites. Nanomaterials.

[43] Naganuma Y., Horiuchi T., Kato C., Tanaka S. (2013). Low-temperature synthesis of silica coating on a poly (ethylene terephthalate) film from perhydropolysilazane using vacuum ultraviolet light irradiation. Surf. Coat. Technol..

[44] Tanaka K., Hanaoka K., Yamaguchi M., Shindo T., Kunzelmann K.H., Teranaka T. (2011). Silica film coating method for veneering resin composite. Dent. Mater. J..

[45] Morlier A., Cros S., Garandet J.P., Alberola N. (2012). Thin gas-barrier silica layers from perhydropolysilazane obtained through low temperature curings: A comparative study. Thin Solid Films.

[46] Khan S., Ahn H.-Y., Han J.S., Ju B.-K., Lee S.Y., Jang H.S., Byun J.Y., Cho S.-H. (2020). Luminescent silica films prepared using perhydropolysilazane and Mn-doped ZnS nanophosphors. Appl. Surf. Sci..

[47] Niizeki T., Nagayama S., Hasegawa Y., Miyata N., Sahara M., Akutsu K. (2016). Structural study of silica coating thin layers prepared from perhydropolysilazane: Substrate dependence and water penetration structure. Coatings.

[48] Burak D., Rahman M.A., Seo D.-C., Byun J.Y., Han J., Lee S.E., Cho S.-H. (2023). In situ metal deposition on perhydropolysilazane-derived silica for structural color surfaces with antiviral activity. ACS Appl. Mater. Interfaces.

[49] Jung S.-H., Lee J.-S., Oh J.-H., Moon S.-W., Kim S.-D. (2010). Method of low-temperature conversion of perhydropolysilazane into amorphous SiO_x_ in aqueous solutions. Jpn. J. Appl. Phys..

[50] Qin H., Wen Y., Liu Q. (2022). Application of SiON coatings in sandstone artifacts conservation. Coatings.

[51] Duo L., Zhang Z., Zheng K., Wang D., Xu C., Xia Y. (2020). Perhydropolysilazane derived SiON interfacial layer for Cu/epoxy molding compound composite. Surf. Coat. Technol..

[52] Hou Y., Yang X., Li B., Li D., Gao S., Wu Z. (2019). Preparation and interface modification of Si_3_N_4f_/SiO_2_ composites. J. Mater. Sci. Technol..

[53] Li D., Yu Q., Li X., Li J., Liu R., Wang Y., Wan F. (2023). Study on the fiber/matrix interface modification of Si_3_N_4f_/SiO_2_ composites with polymer derived double-layer coatings. J. Eur. Ceram. Soc..

[54] Shmagina E., Volobujeva O., Nasibulin A.G., Bereznev S. (2024). Fabrication of novel SiO_x_N_y_/SWCNT laminate-type composite protective coating using low-temperature approach. Ceram. Int..

[55] Shmagina E., Antonov M., Kasikov A., Volobujeva O., Khabushev E.M., Kallio T., Bereznev S. (2024). Structural, mechanical, and optical properties of laminate-type thin film SWCNT/SiO_x_N_y_ composites. Nanomaterials.

[56] Yokoyama Y., Horiuchi K., Maeshima Ohta T. (1994). X-ray photoelectron spectroscopy and X-ray absorption near edge structure study of structural change of perhydropolysilazane to silicon nitride by heat treatment. Jpn. J. Appl. Phys..

[57] Je S.Y., Son B.-G., Kim H.-G., Park M.-Y., Do L.-M., Choi R., Jeong J.K. (2014). Solution-processable LaZrO_x_/SiO_2_ gate dielectric at low temperature of 180 °C for high-performance metal oxide field-effect transistors. ACS Appl. Mater. Interfaces.

[58] Seul H.J., Kim H.G., Park M.Y., Jeong J.K. (2016). A solution-processed silicon oxide gate dielectric prepared at a low temperature *via* ultraviolet irradiation for metal oxide transistors. J. Mater. Chem. C.

[59] Kobayashi Y., Yokota H., Fuchita Y., Takahashi A., Sugahara Y. (2013). Characterization of gas barrier silica coatings prepared from perhydropolysilazane films by vacuum ultraviolet irradiation. J. Ceram. Soc. Jpn..

[60] Baek J.J., Park S.M., Kim Y.R., Chang K.C., Heo Y.-J., Bae G.Y., Choi K.H., Shin G. (2022). Intense pulsed UV light treatment to design functional optical films from perhydropolysilazane: An alternative to conventional heat treatment processes. J. Mater. Sci..

[61] Zhan Y., Grottenmüller R., Li W., Javaid F., Riedel R. (2021). Evaluation of mechanical properties and hydrophobicity of room-temperature, moisture-curable polysilazane coatings. J. Appl. Polym. Sci..

[62] Kubo T., Tadaoka E., Kozuka H. (2004). Preparation of hot water-resistant silica thin films from polysilazane solution at room temperature. J. Sol.-Gel Sci. Technol..

[63] Ohishi T., Sone S., Yanagida K. (2014). Preparation and gas barrier characteristics of polysilazane-derived silica thin films using ultraviolet irradiation. Mater. Sci. Appl..

[64] Ohishi T., Ichikawa K. (2019). Formation and gas barrier properties of silica thin films formed on heat-resistant PET (Polyethylene Terephthalate) substrate by excimer light irradiation to polysilazane coatings. Mater. Lett..

[65] Smokovych I., Bolbut V., Krüger M., Scheffler M. (2019). Tailored oxidation barrier coatings for Mo-Hf-B and Mo-Zr-B alloys. Materials.

[66] Yue S., Wang S., Han D., Huang S., Xiao M., Meng Y. (2022). Perhydropolysilazane-derived-SiO_x_ coated cellulose: A transparent biodegradable material with high gas barrier property. Cellulose.

[67] Ohishi T. (2003). Gas barrier characteristics of a polysilazane film formed on an ITO-coated PET substrate. J. Non-Cryst. Solids.

[68] Lee J.Y., Saito R. (2016). Transparency and water vapor barrier properties of polybenzoxazine-silica nanocomposites provided with perhydropolysilazane. J. Appl. Polym. Sci..

[69] Saito R., Hosoya T. (2008). Water vapor barrier property of organic–silica nanocomposite derived from perhydropolysilazane on polyvinyl alcohol substrate. Polymer.

[70] Lee J.Y., Takeichi T., Saito R. (2016). Study on synthesis and the reaction mechanism of polybenzoxazine-silica nanocomposites provided from perhydropolysilazane. Polymer.

[71] Xue J. (2026). The stability turn in perovskite photovoltaics. Nat. Rev. Mater..

[72] Patiño López J.J., Rivera Botia K.G., Ballestas K., Velilla E., Montoya J.F., Jaramillo F., Ramirez D. (2026). The long road to outdoor stability: Real-world challenges for controlling perovskite materials for solar cells. Chem. Soc. Rev..

[73] Tanko K.T., Tian Z., Raga S., Xie H., Katz E.A., Lira-Cantu M. (2025). Stability and reliability of perovskite photovoltaics: Are we there yet?. MRS Bull..

[74] Nur-E-Alam M., Islam M.S., Abedin T., Islam M.A., Yap B.K., Kiong T.S., Das N., Rahman M.R., Khandaker M.U. (2025). Current scenario and future trends on stability issues of perovskite solar cells: A mini review. Curr. Opin. Colloid Interface Sci..

[75] Yang Y., Song X., Liu H., Zhang A., Cao J., Wu C. (2025). Encapsulation-driven stability in perovskite solar cells: Suppressing degradation through hermetic sealing. ACS Appl. Mater. Interfaces.

[76] Nawaz M.H., Mai T.-H., Seok S.I.I., Chuang F.-C., Pham P.V., Park N.-G. (2025). Flexible perovskite solar cells: Advancements in materials, fabrication techniques, and future prospects. Nano Converg..

[77] Singh T., Miyasaka T. (2018). Stabilizing the efficiency beyond 20% with a mixed cation perovskite solar cell fabricated in ambient air under controlled humidity. Adv. Energy Mater..

[78] Niu G., Guo X., Wang L. (2015). Review of recent progress in chemical stability of perovskite solar cells. J. Mater. Chem. A.

[79] Wang R., Mujahid M., Duan Y., Wang Z.-K., Xue J., Yang Y. (2019). A review of perovskites solar cell stability. Adv. Funct. Mater..

[80] Kim J., Jang J.H., Kim J.-H., Park K., Jang J.S., Park J., Park N. (2020). Inorganic encapsulation method using solution-processible polysilazane for flexible solar cells. ACS Appl. Energy Mater..

[81] Bharadwaj R.K. (2001). Modeling the barrier properties of polymer-layered silicate nanocomposites. Macromolecules.

[82] Ray S.S., Okamoto M. (2003). Polymer/layered silicate nanocomposites: A review from preparation to processing. Prog. Polym. Sci..

[83] Sanchez C., Belleville P., Popall M., Nicole L. (2011). Applications of advanced hybrid organic–inorganic nanomaterials: From laboratory to market. Chem. Soc. Rev..

[84] Morlier A., Cros S., Garandet J.-P., Alberola N. (2013). Gas barrier properties of solution processed composite multilayer structures for organic solar cells encapsulation. Sol. Energy Mater. Sol. Cells.

[85] Mehta S., Sheng H., Krishnan R., Haran B., Han T., Bayindir Z., Berardi M., Yatzor B., Liu J., Shepard J. (2018). UV assisted densification of perhydropolysilazane (PHPS) based spin-on glass in high aspect ratio gap fill structure. ECS Trans..

[86] Saito R. (2006). Synthesis and properties of organic-silica nanocomposites with perhydropolysilazane. J. Polym. Sci. Part A Polym. Chem..

[87] Shin Y.J., Kim H.G., Choi S.Y., Kim S.M., Kang J.E., Han H.W., Kim J.M., Kim G.H., Yeom G.Y. (2024). Effect of bias frequency on bottom-up SiO_2_ gap-filling using plasma-enhanced atomic layer deposition. ACS Appl. Mater. Interfaces.

[88] Aritome S., Kikkawa T. (2013). Scaling challenge of Self-Aligned STI cell (SA-STI cell) for NAND flash memories. Solid-State Electron..

[89] Talukdar T.K., Wang W.B., Girolami G.S., Abelson J.R. (2018). Superconformal coating and filling of deep trenches by chemical vapor deposition with forward-directed fluxes. J. Vac. Sci. Technol. A.

[90] Santorelli M., Tortai J.H., Querré M., Bonvalot M. (2026). Selective plasma enhanced atomic layer deposition for deep trench filling: A design-of-experiments approach. Chem. Eng. Res. Des..

[91] Burak D., Han J.H., Han J.S., Kim I.S., Rahman M.A., Yang J.K.W., Cho S.-H. (2024). Controlling TiO_2_ photocatalytic behaviour *via* perhydropolysilazane-derived SiO_2_ ultrathin shell. Nanoscale.

[92] Yamano A., Kozuka H. (2009). Preparation of silica coatings heavily doped with spiropyran using perhydropolysilazane as the silica source and their photochromic properties. J. Phys. Chem. B.

[93] Akutsu-Suyama K., Kira H., Miyata N., Hanashima T., Miyazaki T., Kasai S., Yamazaki D., Soyama K., Aoki H. (2020). Fine-structure analysis of perhydropolysilazane-derived nano layers in deep-buried condition using polarized neutron reflectometry. Polymers.

[94] Akutsu K., Cagnes M., Niizeki T., Hasegawa Y., Darwish T.A. (2018). Penetration behavior of an ionic liquid in thin-layer silica coating: Ionic liquid deuteration and neutron reflectivity analysis. Phys. B Condens. Matter.

[95] Günthner M., Schütz A., Glatzel U., Wang K., Bordia R.K., Greißl O., Krenkel W., Motz G. (2011). High performance environmental barrier coatings, Part I: Passive filler loaded SiCN system for steel. J. Eur. Ceram. Soc..

[96] Smokovych I., Krüger M., Scheffler M. (2019). Polymer derived ceramic materials from Si, B and MoSiB filler-loaded perhydropolysilazane precursor for oxidation protection. J. Eur. Ceram. Soc..

[97] Seifert M., Motz G. (2016). Synthesis and high-temperature oxidation of a polymer-derived Mo-SiN based ceramic composite. J. Eur. Ceram. Soc..

[98] Smokovych I., Hasemann G., Krüger M., Scheffler M. (2017). Polymer derived oxidation barrier coatings for Mo-Si-B alloys. J. Eur. Ceram. Soc..

[99] Monti M., Dal Bianco B., Bertoncello R., Voltolina S. (2008). New protective coatings for ancient glass: Silica thin-films from perhydropolysilazane. J. Cult. Herit..

[100] Chen Q., Zhang X., Wang F. (2021). Experimental study of thermal diffusion enhanced vapor transfer performance with perhydropolysilazane-derived silica (PDS) coating membranes in air dehumidification process. Int. J. Refrig..

[101] Song J., Wang D., Hu L., Huang X., Chen Y. (2018). Superhydrophobic surface fabricated by nanosecond laser and perhydropolysilazane. Appl. Surf. Sci..

[102] Iwamoto Y., Sato K., Kato T., Inada T., Kubo Y. (2005). A hydrogen-permselective amorphous silica membrane derived from polysilazane. J. Eur. Ceram. Soc..

[103] Mohd Sokri M.N., Daiko Y., Mouline Z., Honda S., Iwamoto Y. (2016). Formation of micro and mesoporous amorphous silica-based materials from single source precursors. Inorganics.

[104] Mohd Sokri M.N., Onishi T., Mouline Z., Daiko Y., Honda S., Iwamoto Y. (2015). Polymer-derived amorphous silica-based inorganic–organic hybrids having alkoxy groups: Intermediates for synthesizing microporous amorphous silica materials. J. Ceram. Soc. Jpn..

[105] Mohd Sokri M.N., Daiko Y., Honda S., Iwamoto Y. (2015). Synthesis of microporous amorphous silica from perhydropolysilazane chemically modified with alcohol derivatives. J. Ceram. Soc. Jpn..

[106] Takeuchi K., Nemoto D., Suga T., Higurashi E. Conversion of perhydropolysilazane into SiO_2_ using plasma treatment for wafer bonding. Proceedings of the 2023 18th International Microsystems, Packaging, Assembly and Circuits Technology Conference (IMPACT).

[107] Esposito Corcione C., Frigione M. (2011). Influence of stone particles on the rheological behavior of a novel photopolymerizable siloxane-modified acrylic resin. J. Appl. Polym. Sci..

[108] Negri A., Nervo M., Marcello S.D., Castelli D. (2021). Consolidation and adhesion of pictorial layers on a stone substrate: The study case of the Virgin with the Child from Palazzo Madama, in Turin. Coatings.

[109] Xin D., Han Q. (2015). Adhesion reliability of the epoxy–Cu interface by molecular simulations. J. Adhes..

[110] Fu S.-W., Lee C.-C. (2017). A corrosion study of Ag–Al intermetallic compounds in chlorine-containing epoxy molding compounds. J. Mater. Sci. Mater. Electron..

